# Color arrestor pixels for high-fidelity, high-sensitivity imaging sensors

**DOI:** 10.1515/nanoph-2024-0064

**Published:** 2024-04-15

**Authors:** Mingwan Cho, Joonkyo Jung, Myungjoon Kim, Jeong Yub Lee, Seokhwan Min, Jongwoo Hong, Shinho Lee, Minsung Heo, Jong Uk Kim, In-Sung Joe, Jonghwa Shin

**Affiliations:** Department of Materials Science and Engineering, 34968 KAIST, Daejeon 34141, Republic of Korea; 65473 Samsung Advanced Institute of Technology, 130, Samsung-ro, Yeongtong-gu, Suwon-si, Gyeonggi-do 16678, Republic of Korea; Semiconductor Research Center, 65665 Samsung Electronics, Samsungjeonja-ro 1, Hwaseong-si, Gyeonggi-do 18448, Republic of Korea

**Keywords:** color matching functions, linear transformation, color fidelity, CMOS image sensor

## Abstract

Silicon is the dominant material in complementary metal-oxide-semiconductor (CMOS) imaging devices because of its outstanding electrical and optical properties, well-established fabrication methods, and abundance in nature. However, with the ongoing trend toward electronic miniaturization, which demands smaller pixel sizes in CMOS image sensors, issues, such as crosstalk and reduced optical efficiency, have become critical. These problems stem from the intrinsic properties of Si, particularly its low absorption in the long wavelength range of the visible spectrum, which makes it difficult to devise effective solutions unless the material itself is changed. Recent advances in optical metasurfaces have offered new possibilities for solving these problems. In this study, we propose color arrestor pixels (CAPs) as a new class of color image sensors whose composite spectral responses directly mimic those of the human eye. The key idea is to employ linearly independent combinations of standardized color matching functions. These new basis functions allow our device to reproduce colors more accurately than the currently available image sensors with red-green-blue filters or other metasurface-based sensors, demonstrating an average CIEDE2000 color difference value of only 1.79 when evaluating 24 colors from the Gretag-Macbeth chart under standard illuminant D65. Owing to their high fidelity to the human eye response, CAPs consistently exhibit exceptional color reproduction accuracy under various spectral illumination compositions. With a small footprint of 860 nm height and 221 nm full-color pixel pitch, the CAPs demonstrated high absorption efficiencies of 79 %, 81 %, and 63 % at wavelengths of 452 nm, 544 nm, and 603 nm, respectively, and good angular tolerance. With such a high density of pixels efficiently capturing accurate colors, CAPs present a new direction for optical image sensor research and their applications.

## 1 Introduction

**Figure 1:**
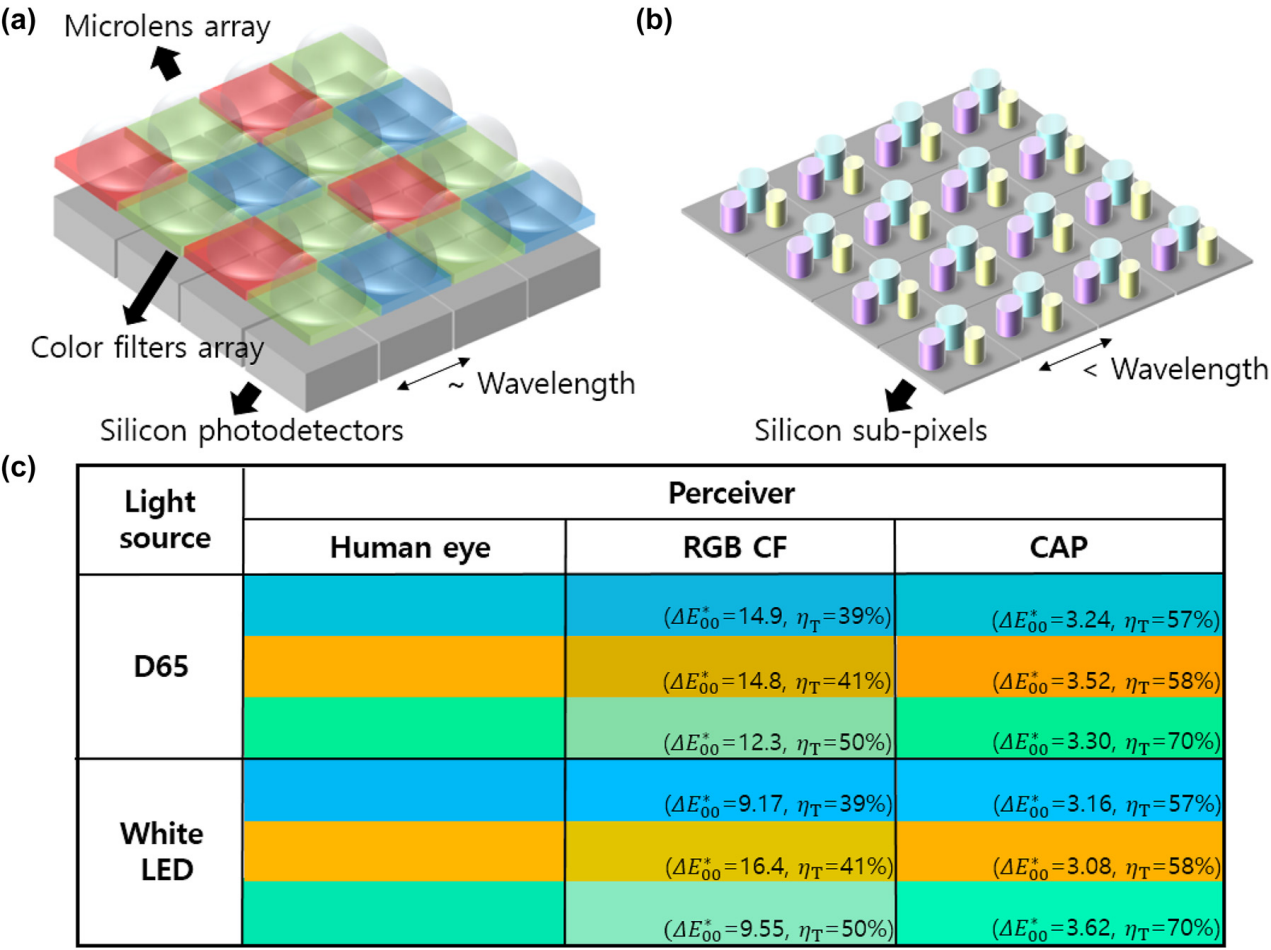
Comparison of conventional CIS with Bayer RGB color filters and CAPs. (a) Schematic illustration of conventional CIS featuring a microlens array, Bayer RGB color filter array, and Si photodetectors. (b) A diagram of the CAPs, with each supercell composed of three different subpixels. (c) A visual comparison of colors among the naked human eye, CIS with Bayer RGB color filters, and CAPs when observed under standard illuminant D65 and white LED. The values within the color boxes of both conventional CIS with Bayer RGB color filters and CAPs represent the color difference value $\left(\Delta E_{00}^{*}\right)$ compared to the color captured by the human eye, as well as the total quantum efficiency (*η*_*T*_) of the device at specific wavelengths.

Optical image sensors are increasingly gaining widespread use in diverse applications, including mobile devices, augmented reality glasses, and sensors for autonomous vehicles and robots. The demand for high-quality image sensors has driven rapid evolution in various aspects, such as increased image resolution [1]–[9], improved low-light imaging capabilities [10]–[12], and pixel miniaturization [13]–[15]. Currently, complementary metal-oxide-semiconductor (CMOS) image sensors (CIS) have mainly utilized silicon (Si)-based photodetectors with additional components, such as color filters [16], [17], microlenses, and antireflection coatings [18], to facilitate color separation and efficient light collection, as illustrated in Figure 1(a). In this configuration, the CIS typically employs color filters with a Bayer pattern layout (or a modified version with pixel binning), and Si photodetectors underneath are divided accordingly. However, this approach has inherent limitations in terms of the optical performance. Specifically, only 25 % of the Si photodetector area is allocated for collecting the red (R) or blue (B) wavelength ranges, whereas 50 % of the Si photodetector area is allocated for green (G). Moreover, the indirect bandgap of Si results in low absorption coefficients for wavelengths longer than 600 nm, making it difficult to achieve satisfactory absorption performance and sensor thicknesses below a few microns [19]. A sufficient thickness is necessary to ensure adequate absorption; however, this raises the issue of light spreading into neighboring pixels. Although the insertion of barriers between Si photodetectors [5], [9] has been suggested to mitigate these limitations, the problems are escalating as the lateral size of the pixels has reached the submicron scale for the current generation of sensors.

Metasurfaces, which are planar arrays of subwavelength nanostructures, may provide viable solutions with the potential to overcome the limitations of existing devices originating from the intrinsic properties of the materials. In particular, through the proper design of nanostructures, one can induce tailored optical resonances that can intensify the electromagnetic fields inside the silicon layer, facilitating efficient absorption in a thin device. Moreover, by tuning the size, shape, and placement of the nanostructures, the frequency-dependent effective surface impedance can be precisely controlled, potentially eliminating the need for separate antireflection layers and color filters. There have been successful demonstrations of replacing individual CIS components with metasurfaces with enhanced properties, such as high-efficiency color filters with enhanced spectral responsivity and resolution [16], [20]. Furthermore, recent studies incorporated inverse design techniques to create high-resolution color routers with improved efficiency [10]–[12]. These color routers employ color-selective routing rather than absorption and can overcome the efficiency limitations of conventional absorptive color filters. However, substituting only a portion of the CIS with a metasurface has limitations in terms of reducing the overall device thickness and simplifying its structure. Hence, recent research has aimed to reduce the overall thickness of devices by simultaneously integrating the functionalities of microlenses, color filters, antireflection coatings, and Si photodetectors into a single metasurface. For example, anti-Hermitian Si metasurface [13], [14], Si–Al hybrid nanoantennas [15], and filter-free image sensor pixels comprising Si nanowire [21] have been proposed as miniaturized CMOS-compatible image sensors. Additionally, advanced design like stacked silicon and germanium nanopillars [22] shows promise for the next generation of compact CMOS image sensors. However, these structures require additional design considerations for their use in actual CIS products. One problem is accurate color reproduction. Most of the previously proposed color-selective metasurfaces were designed to maximize absorption in three specific wavelength ranges (620–750 nm for red, 500–565 nm for green, and 450–485 nm for blue) while minimizing crosstalk from other wavelength ranges. However, it can be mathematically proven that color reproduction based on this approach cannot generate the same colors as captured by the human eye under all lighting conditions because the spectral response functions of the three types of cone cells in the human retina overlap with one another. Another issue is incident-angle dependency. Ideally, the measured colors should not change when the same light is incident from different angles; however, many metasurfaces exhibit strong angle dependency in their spectral absorption.

In this study, we first propose color arrestor pixels (CAPs), whose spectral absorptances can be represented as linearly independent combinations of normalized color matching functions (NCMFs), as a fundamental solution to guarantee color accuracy under all lighting conditions. CAPs are based on supercell structures, as schematically shown in Figure 1(b). Each supercell incorporated three subpixels with distinct designs, and the spectral absorptance of each subpixel was optimized for representation as a distinctive linear combination of NCMFs. Based on this approach, CAPs demonstrate significant light absorption capabilities at peak absorption wavelengths while maintaining a compact form factor. This approach enables a reduction in pixel pitch and device height by eliminating the need for separate color filters or color routers atop Si photodetectors, which typically require some propagation distances. Furthermore, the CAPs exhibited accurate color reproduction akin to that of the naked human eye under diverse illuminating light sources, such as the standard illuminant D65 and white LED, as illustrated in Figure 1(c). Compared to conventional CIS with Bayer red-green-blue (RGB) color filters, which assume that incident light passes through the microlens array without loss and is completely absorbed by a sufficiently thick Si photodetector, the CAPs outperform in both visual color comparisons and efficiency aspects. Furthermore, we demonstrate the high performance of CAPs in terms of angle tolerance.

## 2 Results

### 2.1 Design principles

The *Commission Internationale de l’éclairage* (CIE) color spaces are standard ways to represent colors in a quantitative and device-independent way. Although there are several versions of CIE color spaces, all are based on a set of three standardized color matching functions (CMFs) that numerically convert the power spectral density of the captured light to a CIE tristimulus value tuple $\left(X,Y,Z\right)$ . The CMFs, namely $\bar{x}\left(\lambda \right)$ , $\bar{y}\left(\lambda \right)$ , and $\bar{z}\left(\lambda \right)$ , are derived based on a scientific model of the human eye’s chromatic response such that they can quantitatively explain how humans capture colors. Therefore, if one can construct three types of photodetectors with response functions mimicking standardized CMFs, an imaging system comprising such detectors would have a color perception very similar to that of humans under any lighting condition.

Metasurfaces can realize a diverse range of absorption spectra through customized optical resonances. Thus, they are natural candidates for designing such “human-like” photodetectors. However, the strong absorption of silicon, an almost exclusive absorber material in visible-light imaging, especially in the blue part of the spectrum, makes the direct implementation of CMFs (particularly, $\bar{y}\left(\lambda \right)$ that should have slight blue absorption) difficult. Instead, we aim to implement three linearly independent combinations of $\bar{x}\left(\lambda \right)$ , $\bar{y}\left(\lambda \right)$ , and $\bar{z}\left(\lambda \right)$ . If this is possible, then a simple multiplication of the inverse matrix can generate the desired $\left(X,Y,Z\right)$ tuple.

Before further explaining the process, we first introduce modified versions of the CMFs, called normalized color matching functions (NCMFs), expressed as

(1) $$\begin{array}{c} {\bar{x}}^{\prime }\left(\lambda \right)=\frac{\lambda_{0}}{\eta \lambda }\bar{x}\left(\lambda \right)\\ \begin{array}{c} {\bar{y}}^{\prime }\left(\lambda \right)=\frac{\lambda_{0}}{\eta \lambda }\bar{y}\left(\lambda \right) \end{array}\\ {\bar{z}}^{\prime }\left(\lambda \right)=\frac{\lambda_{0}}{\eta \lambda }\bar{z}\left(\lambda \right) \end{array}$$

where ${\bar{x}}^{\prime }\left(\lambda \right),{\bar{y}}^{\prime }\left(\lambda \right)$ , and ${\bar{z}}^{\prime }\left(\lambda \right)$ are NCMFs corresponding to the standardized CMFs, $\bar{x}\left(\lambda \right)$ , $\bar{y}\left(\lambda \right)$ , and $\bar{z}\left(\lambda \right)$ , respectively, and *η* refers to the internal quantum efficiency of the photodetector. Moreover, *λ*_0_ is a reference wavelength (which can be set to an arbitrary value such as 532 nm), and *λ* is the wavelength of light. This modification reflects the actual conversion process in silicon photodetectors: an absorbed photon generates an electron–hole pair that results in detectable signals with probability *η*. The inverse scaling relationship to the wavelength is because the energy of a single photon is inversely proportional to its wavelength. Because the actual output signal of the photodetectors as a function of the input wavelength should be a combination of the original color matching functions, the power absorptance of the photodetectors as a function of the wavelength should also be a combination of NCMFs. Specifically, more power should be absorbed at shorter wavelengths to generate the correct spectral responses.

**Figure 2:**
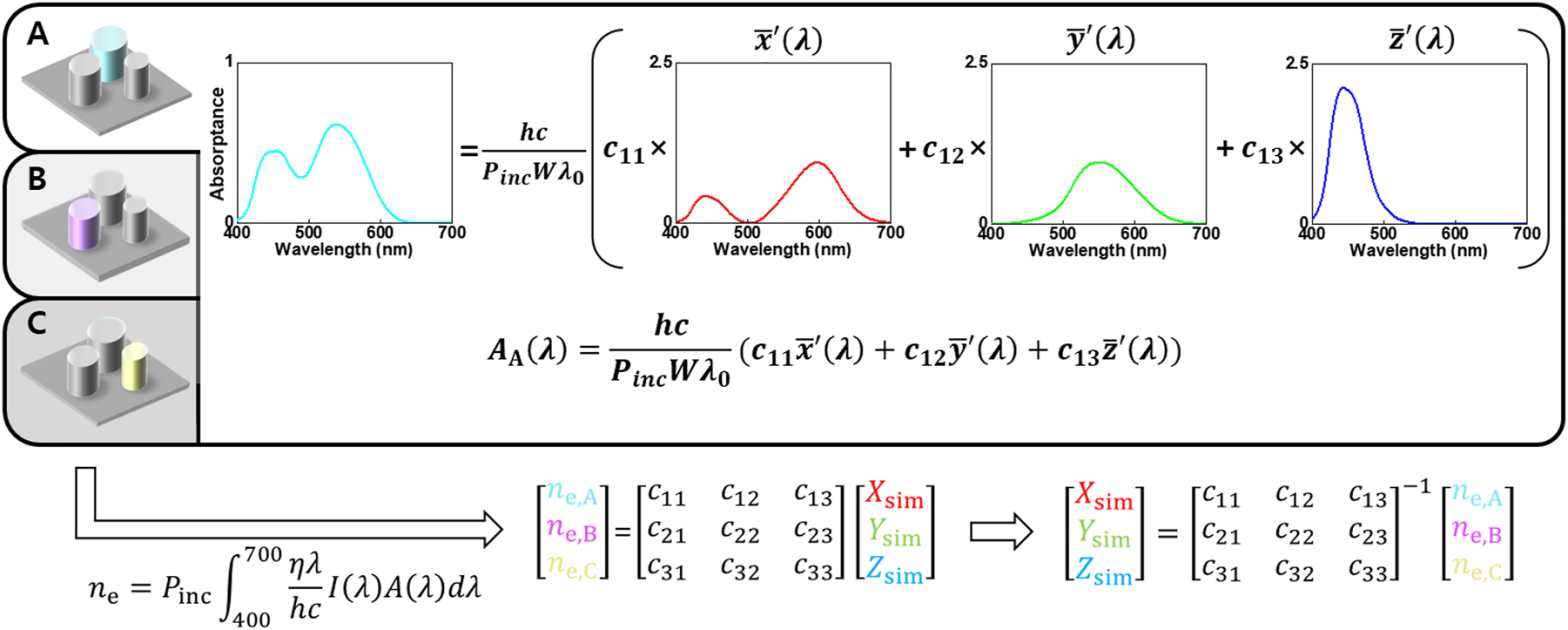
Fundamental concept of ideal CAPs. It consists of three different subpixels within a single supercell. The spectral absorptance of each subpixel can be represented as a linear combination of NCMFs. By using the relationship between the spectral absorptance of subpixels *A*(*λ*) and the number of detected electrons in the CAPs *n*_*e*_, the color extraction process can be represented as a simple matrix multiplication.

As conceptually shown in Figure 2, the proposed CAPs consist of three subpixels within a single supercell, each with a distinct spectral absorptance. The subpixels may be on the same plane or in a stacked configuration, as discussed later. The output signal of each subpixel is expressed as

(2) $$\begin{array}{c} n_{e,i}=P_{\text{inc}}\int_{400}^{700}\frac{\eta \lambda }{hc}R\left(\lambda \right)I\left(\lambda \right)A_{i}\left(\lambda \right)\text{d}\lambda \end{array}$$

where *n*_*e*,*i*_ is the number of detected electrons in the subpixel *i* (*i* = A, B, and C), *P*_inc_ is the power of incident light on a super-pixel if the object is perfectly reflecting, *h* is the Planck constant, *c* is the speed of light, $R\left(\lambda \right)$ is the spectral reflectance (or transmittance) of the imaged object, *I*(*λ*) is the normalized spectral power distribution function of the illuminant, and $A_{i}\left(\lambda \right)$ is spectral absorptance of the subpixel *i* (see Supplementary Note 1 for the detailed derivation). We set the wavelength range from 400 nm to 700 nm for spectral integration. In terms of material properties, silicon photodetectors can absorb light outside the above wavelength range as well. In comparison, our eyes (color matching functions) are insensitive at wavelengths below 400 nm and above 700 nm. Hence, using the light outside the above wavelength range does not aid in obtaining color accurate vision within the visible spectrum and blur the images due to chromatic aberration of lenses. Therefore, we have assumed the presence of UV and IR-cut filters in the lens system in our study to improve color accuracy and image sharpness. Thus, only the visible light spectrum of 400–700 nm is allowed to pass through.

Transitioning to spectral absorptance analysis, if the spectral absorptance *A*_*i*_(*λ*) is a linear combination of NCMFs, as shown in Figure 2, where *c*_*i*1_, *c*_*i*2_, and *c*_*i*3_ are the coefficients, *n*_*e*,*i*_ can be expressed as a linear combination of tristimulus values with the same coefficients, expressed as *n*_*e*,*i*_ = *c*_*i*1_ *X*_sim_ + *c*_*i*2_ *Y*_sim_ + *c*_*i*3_ *Z*_sim_ (see Supplementary Note 2 for details). By representing the number of detected electrons in the subpixels and the correct tristimulus values of the particular situation as column vectors, their relationship can be conveniently expressed in the form of a matrix-vector multiplication, as shown in Figure 2. In particular, the tristimulus values can be extracted in a straightforward manner by simply multiplying the inverse of the coefficient matrix by the vector of the number of detected electrons in each subpixel.

### 2.2 Design of CAPs

**Figure 3:**
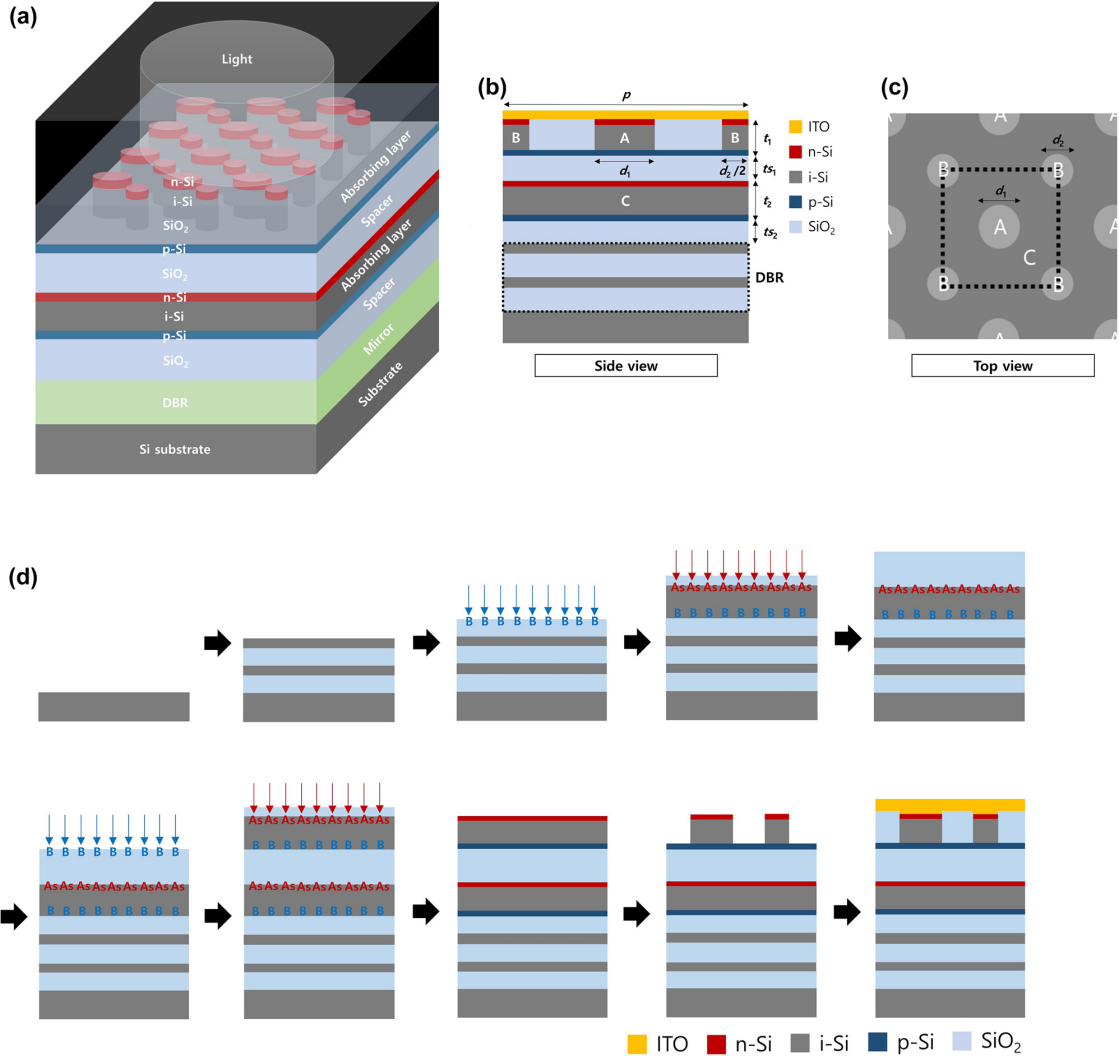
Design of CAPs. (a) Schematic illustration of the designed CAPs showing two absorbing layers and the distributed Bragg reflector (DBR) layers. (b) Side view of a CAPs unit cell design composed of Si nanodisk arrays in the upper active layer and an unpatterned Si slab in the lower active layer with p-i-n junctions, separated by SiO_2_ spacers on the DBR. (c) Top view of the CAPs design. The Si nanodisk arrays are arranged in a checkerboard pattern. Each type of nanodisk and an unpatterned Si slab are responsible for each subpixel. (d) Simplified version of possible sample preparation processes (formation of via connections is omitted).

Among the many possible designs for CAPs, we chose a double-layered configuration as an example, as shown in Figure 3. In this design, subpixels A and B were positioned on the upper Si layer, and subpixel C was positioned on the lower Si layer. This configuration was adopted because silicon has considerably smaller absorption coefficients in the red part of the visible spectrum than in blue or even green, which has thus far hindered the miniaturization of image sensor pixels in the industry. The readily absorbed blue and green light can be efficiently harvested by subpixels A and B with smaller volumes, whereas the problematic red light can be mostly absorbed by subpixel C on the lower layer with the help of the distributed Bragg mirror (DBR) layer beneath. Alternatively, one could choose a single-layer configuration, which would potentially be easier to fabricate, albeit with some sacrifice in the absorption efficiency, as shown in Figure S1 of the Supplementary Material. Figure 3(a) depicts a distributed Bragg reflector composed of alternating layers of Si and SiO_2_, which primarily reflect the yellow and red parts of the spectrum (see Supplementary Material and Figure S2 for details). The silicon layers in the back reflector were spaced away from the lower absorbing layer by a SiO_2_ spacer with an optimized thickness to ensure maximal absorption of red light through constructive interference. An additional SiO_2_ spacer is placed between the upper and lower active layers to achieve electrical isolation. Each active layer was designed to form a p-i-n junction, where the thicknesses of the n- and p-doped regions were set to 10 nm for effective carrier transportation and collection. Within the upper active layer, arrays of two types of Si nanodisks with different diameters corresponding to subpixels A and B were arranged in a checkerboard pattern. In the lower active layer, an unpatterned Si slab is utilized to construct subpixel C. Because of the C_4_ rotational symmetry, our device can operate regardless of the polarization state of the incident light under normally illuminated [23]. The photocurrent generated from the absorption in subpixel C can be easily obtained using the n-doped and p-doped regions of the lower active layer as electrodes. For each subpixel of the upper active layer, in an isolated manner with SiO_2_, the p-doped region of the upper active layers is assumed to be the electrical common ground for both subpixels, and individual ITO connections to the respective subpixels can be considered, ensuring ohmic contact with n-doped Si and enabling the desired current flow within the device. In addition, we also conceived alternative CAPs designs for individual pixel addressing (see Figures S3 and 4 of the Supplementary Material). With this configuration, the photocurrents generated from the absorption in subpixels A and B, as well as subpixel C, can be independently obtained. Even with the contiguous slab geometry for subpixel C, pixel-by-pixel signals can be obtained simply by attaching individual contacts to each subpixel using the finite diffusion distance of the electrons. Some crosstalk between adjacent super-pixels would not be detrimental to image quality because the super-pixels are considerably smaller than the wavelength in their lateral sizes, which satisfies the Nyquist limit in typical imaging systems. If further isolation is desired, a trench isolation structure can be incorporated into the lower active layer to isolate each super-pixel without significantly altering its spectral absorption properties (see Figure S5 of the Supplementary Material). This design demonstrates the potential applicability of the proposed device to color image sensors. The refractive index data for each material were measured experimentally using an ellipsometer, and the corresponding data are presented in Figure S6 of the Supplementary Material. Possible sample preparation processes for CAPs are illustrated in Figure 3(d) with a detailed explanation provided in the Section 4.

Based on the design of the aforementioned CAPs, we optimized their dimensions using finite-difference time-domain simulations and the particle swarm optimization algorithm. Because only the electrons and holes generated in the intrinsic Si region effectively contribute to the measured photocurrent, the absorption within the volumes of intrinsic Si was included in the evaluation of optical performance. To optimize the CAPs, we considered the following structural parameters as design variables: the thicknesses of the upper and lower Si-absorbing layers $\left(t_{1},t_{2}\right)$ , thicknesses of the two SiO_2_ spacers $\left(ts_{1},ts_{2}\right)$ , period of the super-pixel (*p*), and diameters of the Si nanodisks $\left(d_{1},d_{2}\right)$ . To determine the optimal structures whose spectral absorptances can be represented as linearly independent combinations of NCMFs, we initially define our objective function *F* for optimization as follows:

(3) $$\begin{array}{ll} F & =\frac{1}{w_{11}\left|c_{11}\right|+w_{12}\left|c_{12}\right|+w_{13}\left|c_{13}\right|}\left|A_{\text{sim},\text{A}}-A_{\text{fit},\text{A}}\right|\\ & \;+\frac{1}{w_{21}\left|c_{21}\right|+w_{22}\left|c_{22}\right|+w_{23}\left|c_{23}\right|}\left|A_{\text{sim},\text{B}}-A_{\text{fit},\text{B}}\right|\\ & \;+\frac{1}{w_{31}\left|c_{31}\right|+w_{32}\left|c_{32}\right|+w_{33}\left|c_{33}\right|}\left|A_{\text{sim},\text{C}}-A_{\text{fit},\text{C}}\right| \end{array}$$

where $A_{\text{sim},\text{A}}\left(\lambda \right),A_{\text{sim},\text{B}}\left(\lambda \right)$ , and $A_{\text{sim},\text{C}}\left(\lambda \right)$ are the simulated spectral absorptances of subpixels A, B, and C, $A_{\text{fit},\text{A}}\left(\lambda \right),A_{\text{fit},\text{B}}\left(\lambda \right)$ , and $A_{\text{fit},\text{C}}\left(\lambda \right)$ are the fitted curves that are linear combinations of NCMFs, *c*_*ij*_ is the coefficients of the linear combinations connecting the spectral absorptances and the NCMFs, and *w*_*ij*_ is the weighting factor for the coefficients. As previously mentioned, color extraction can be implemented simply using the inverse matrix of the coefficient matrix. However, if the coefficient matrix is close to an ill-conditioned matrix, its inverse matrix of the coefficient matrix becomes unreliable. Although it is best to make the three row vectors orthogonal to each other, this is challenging because of the broad absorption spectrum for the short wavelength range in the visible spectrum of Si. To prevent the coefficient matrix from becoming ill-conditioned, we introduced weighting factors to allow the three row vectors of the coefficient matrix to differ. We selected the weighting factors (1, 5, 5), (1, 1, 10), and (10, 1, 1) to maximize the inner products between the row vectors. Using these weighting factors, we successfully obtained linearly independent row vectors of the coefficient matrix through optimization. However, the focus of optimization thus far has been on designing Si photodetectors with specific spectral absorptances. The ultimate goal of our CAPs approach is to ensure that any combination of light sources and spectral reflectances can provide the same $\left(X,Y,Z\right)$ tristimulus values as perceived by the human eye. For ideal CAPs, color extraction can be simply implemented through matrix-vector multiplications using the coefficients obtained in the previous section. However, the spectral absorptances of the actual CAPs deviated from the perfect linear combinations of the NCMFs. Thus, the optimization process should target minimization of color errors resulting from this discrepancy. Therefore, the conversion matrix, which maps the output signals from three subpixels of CAPs to the tristimulus values, was considered with 72 cases, encompassing the 24 colors of the Gretag-Macbeth color chart under three different light sources: standard illuminant D65, incandescent, and white LED (see Supplementary Notes 3 and 4 for a detailed explanation of the conversion) and optimized with new objective function *F* as follows:

(4) $$F=\frac{\overset{72}{\underset{i=1}{\sum }}\Delta E_{00}^{*}\left(i\right)}{72}$$

where $\Delta E_{00}^{*}$ is the CIEDE2000 color difference metric. In new optimization (which means optimization of the conversion matrix during structural optimization), we reset the range of geometric parameters near the result derived from initial optimization, and it was optimized to minimize the average color difference across 72 data sets, aiming for high color reproduction as close as possible to human vision. After this optimization, we obtained final values of geometrical parameters as follows: *p* = 221 nm, *t*_1_ = 144 nm, *t*_2_ = 156 nm, *d*_1_ = 117 nm, *d*_2_ = 76 nm, *ts*_1_ = 102 nm, and *ts*_2_ = 118 nm. In addition, we include the performance of the design optimized using only the color matching functions: the results are not as good as those from the above design but still reasonably good.

### 2.3 Optical properties

**Figure 4:**
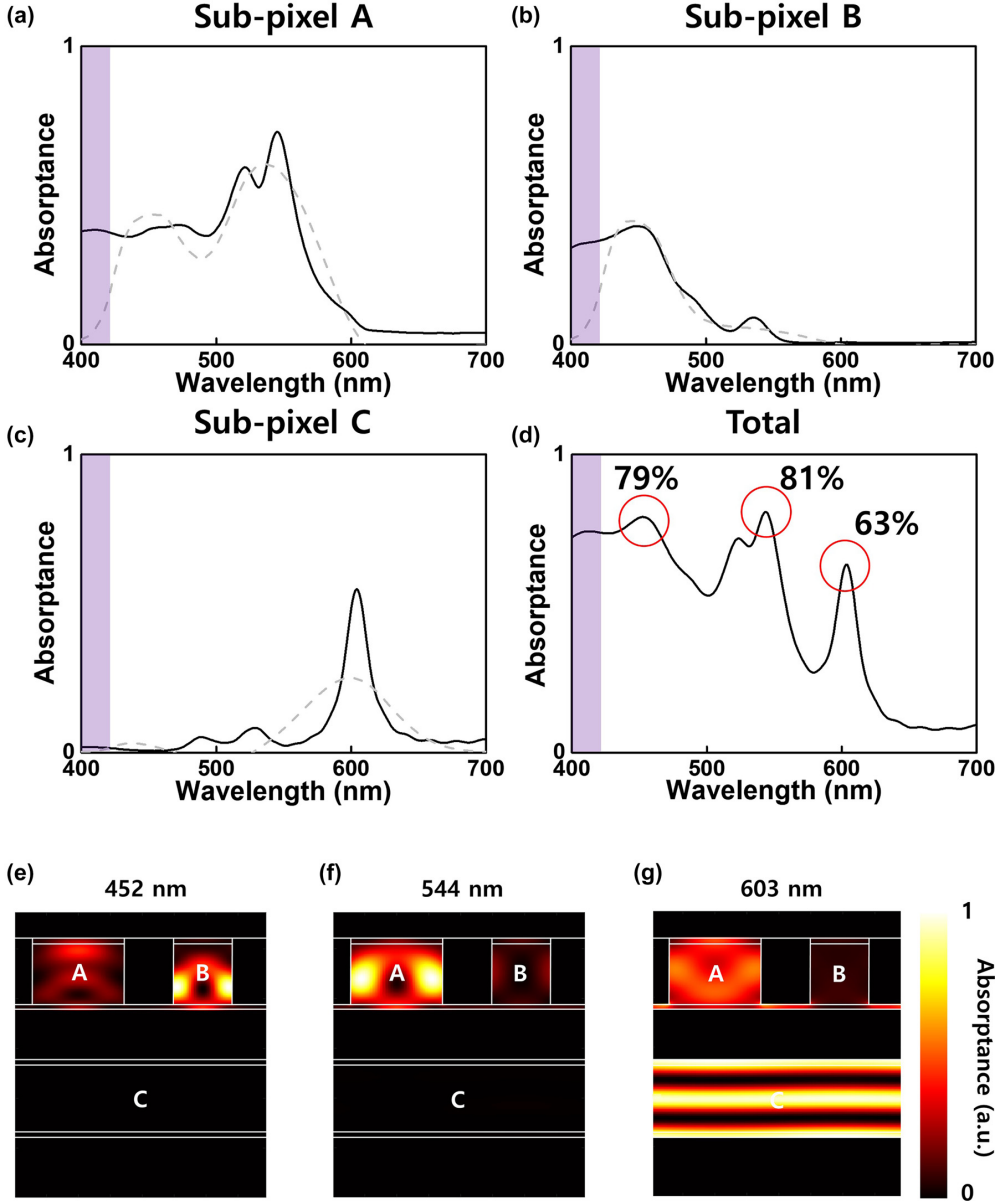
Numerical analysis of optimized CAPs. (a)–(c) The spectral absorptances of subpixels A, B, and C in the CAPs. The black solid lines represent the simulated spectral absorptances of subpixels A, B, and C $\left(A_{\text{sim},\text{A}}\left(\lambda \right),A_{\text{sim},\text{B}}\left(\lambda \right),A_{\text{sim},\text{C}}\left(\lambda \right)\right)$ under linearly polarized light at normal incidence, obtained from finite-difference time-domain simulation. The gray dashed lines represent the curve-fitted spectral absorptances of the subpixels A, B, and C $\left(A_{\text{fit},\text{A}}\left(\lambda \right),A_{\text{fit},\text{B}}\left(\lambda \right),A_{\text{fit},\text{C}}\left(\lambda \right)\right)$ , which are linear combinations of the NCMFs to resemble $\left(A_{\text{sim},\text{A}}\left(\lambda \right),A_{\text{sim},\text{B}}\left(\lambda \right),A_{\text{sim},\text{C}}\left(\lambda \right)\right)$ . (d) The total spectral absorptance of the CAPs. The shaded region indicates the presence of an ultraviolet (UV) filter (400–420 nm) assumed to be located on top of the CAPs. (e–g) The normalized absorption profiles of the unit cell cross section at the peak absorption wavelengths of 452 nm, 544 nm, and 603 nm, respectively.

We determined the optical properties and predicted the read-out values of the optimized CAP structures using finite-difference time-domain simulations. For the analysis, we defined the absorptance as the ratio of the energy absorbed within each subpixel to the incident energy on the entire super-pixel. Figure 4(a)–(c) show the simulated spectral absorptances of subpixels A, B, and C (black solid lines) at normal incidence, along with the fitted curves (gray dashed lines), which are linear combinations of NCMFs. The spectral absorptances in the wavelength range of 400–420 nm were ignored, assuming the presence of an ultraviolet (UV) filter (shaded region in Figure 4(a)–(d)), which is often used in imaging systems to enhance color accuracy. Figure 4(d) shows the total spectral absorptance, represented as a simple sum of the spectral absorptances of individual subpixels, which can be justified by the definition of the spectral absorptances of the subpixels.

The CAPs exhibited considerably high absorption performance of 79 %, 81 %, and 63 % at 452 nm, 544 nm, and 603 nm, respectively, providing an advantage for imaging under low-light conditions. Note that typical red-green-blue color-filter–based image sensors show significantly lower efficiency (Figure 1) because each primary color is mostly utilized in the corresponding subpixel only and is lost in other subpixels. In contrast, the subwavelength nature of the CAP super-pixels allows each subpixel to harvest light from the entire super-pixel. Figure 4(e)–(g) illustrate the normalized absorption profiles along the vertical cross section of the unit cell at peak absorption wavelengths of 452 nm, 544 nm, and 603 nm under linearly polarized light under normal incidence conditions (see Section 4 for calculations). As expected from the aforementioned values of the coefficient matrix, Figure 4(e) illustrates that subpixels A and B exhibited absorption at 452 nm, whereas subpixel C did not. For the green part of the spectrum, Figure 4(f) shows that subpixel A demonstrates strong absorption, subpixel B shows minor absorption, and subpixel C barely absorbs light at 544 nm. Similarly, Figure 4(g) indicates that subpixel C absorbs significantly at 603 nm, in contrast to the minimal absorption observed in subpixel A and negligible absorption in subpixel B. A detailed description of the light absorption properties of the designed subpixels is provided in Supplementary Note 6. In addition, the spectral reflectance of the CAPs was calculated and included in Figure S7 of the Supplementary Material. The CAPs exhibited low reflectance in the wavelength range of 400–600 nm. However, the CAPs demonstrate notable light reflection at wavelengths above 600 nm, which can introduce noise such as flare phenomena in photographs, posing issues for image sensors. In the Supplementary Note 7, we quantitatively calculated and compared the flare between CAPs and CIS with RGB color filters. It was observed that CAPs may have some flares, but not as much as the raw reflection numbers suggest, due to the small absorption of CAPs at wavelengths above 600 nm. Reducing the cut-off wavelength of IR-cut filter down to 650 nm further mitigates the flare problem without noticeable reduction of sensitivity or color accuracy.

### 2.4 Color accuracy

**Figure 5:**
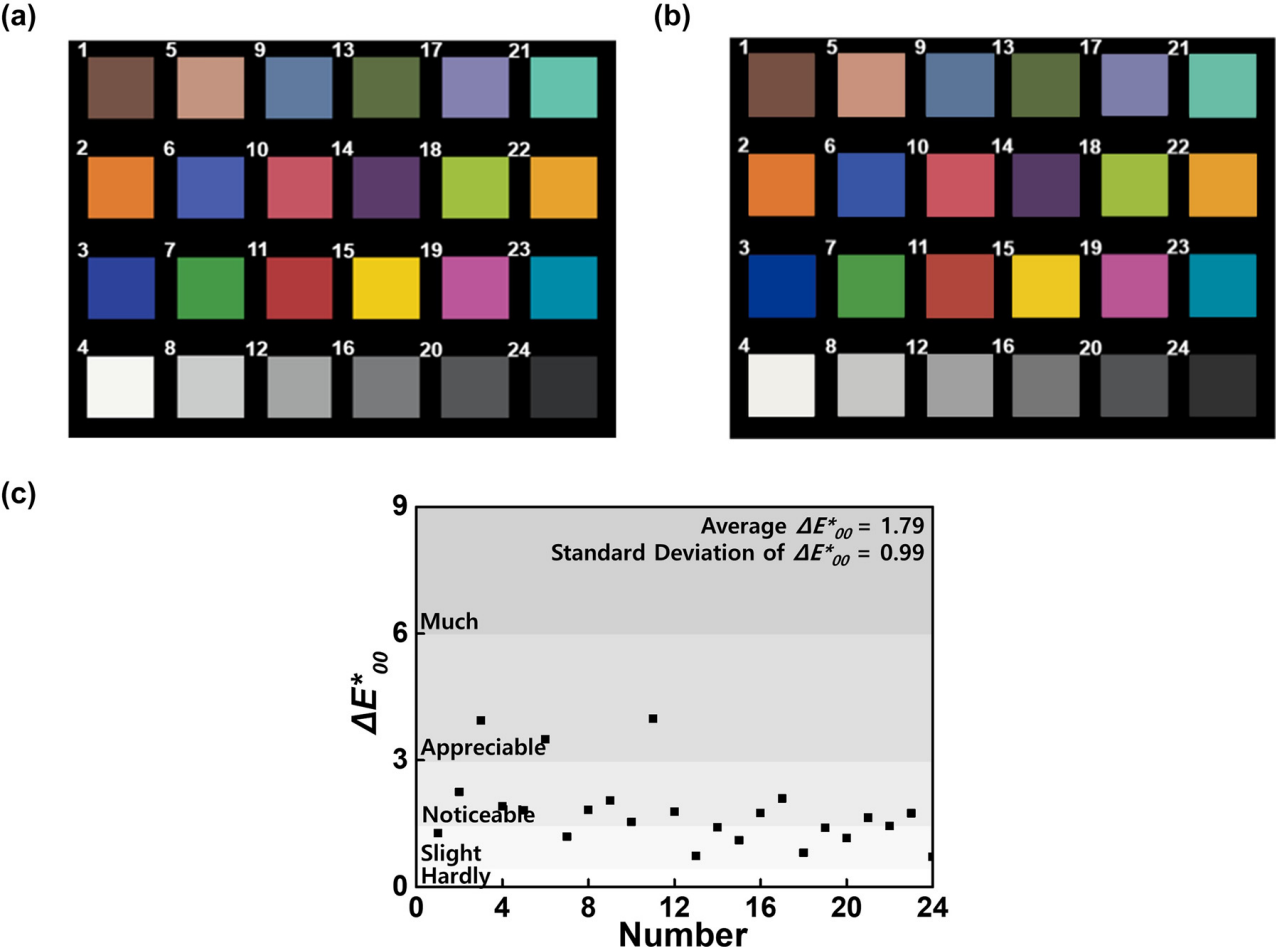
Color comparison. (a) The Gretag-Macbeth color chart under standard illuminant D65 2° observer captured by the naked human eye. (b) Image captured by CAPs of the Gretag-Macbeth color chart under standard illuminant D65 2° observer. (c) The color difference values $\left(\Delta E_{00}^{*}\right)$ for each of the 24 colors of the Gretag-Macbeth color chart by CAPs, employing the CIEDE2000 color difference metric.

To assess the accuracy of color reproduction, we calculated the quantitative color difference between the colors reproduced by CAPs and those captured by human eyes under various illumination sources. The Gretag-Macbeth color chart, which contains 24 distinct colors, was adopted as a reference for color comparison. Firstly, the true tristimulus values representing the colors humanly visible were calculated using the standard formulas: $X_{t}=\frac{1}{W}\int_{400}^{700}R\left(\lambda \right)I\left(\lambda \right)\bar{x}\left(\lambda \right)\text{d}\lambda$ , $Y_{t}=\frac{1}{W}\int_{400}^{700}R\left(\lambda \right)I\left(\lambda \right)\bar{y}\left(\lambda \right)\text{d}\lambda$ , and $Z_{t}=\frac{1}{W}\int_{400}^{700}R\left(\lambda \right)I\left(\lambda \right)\bar{z}\left(\lambda \right)\text{d}\lambda$ . The spectral reflectance $R\left(\lambda \right)$ of each color patch of the Gretag-Macbeth color chart was experimentally measured using UV-visible spectrophotometer. In the case of a conventional CIS with RGB color filters, whose typical spectral transmittance is shown in Figure S8 of the Supplementary Material, the calculation of the tristimulus values was performed using a conversion matrix numerically optimized for the same 72 cases for fair comparison. For a visual demonstration of the colors reproduced from different systems, we converted the CIE color space into the sRGB color space, as shown in Figure 5(a) and (b) (see Supplementary Note 8 for detailed calculations). In cases where the colors in the Gretag-Macbeth color chart fell outside the sRGB color space range, a clipping process was applied [24]. Note that the clipping process is used to make the colors appear within the sRGB color space range that most displays can reproduce; however, the quantitative color difference is accurately calculated without the clipping process, as explained next.

For quantitative comparisons, we employed the CIEDE2000 color difference metric $\left(\Delta E_{00}^{*}\right)$ (see Supplementary Notes 9 and 10 for detailed calculation). $\Delta E_{00}^{*}$ was calculated for each color patch in the Gretag-Macbeth color chart, and the average $\Delta E_{00}^{*}$ was 1.79 with a standard deviation of 0.99 for CAPs under standard illuminant D65, as plotted in Figure 5(c). This result, obtained without any further free-parameter adjustment or postprocessing, indicates that the color reproduction of our device is accurate (see Table S3 of the Supplementary Material for the relationship between $\Delta E_{00}^{*}$ values and typical subjective assessment). Furthermore, we have considered sensor noise, including readout noise and shot noise, which inevitably occurs in actual applications (see Supplementary Note 11 for details). Even in the presence of such noises, CAPs have demonstrated superior color reproduction compared to conventional CIS with RGB color filters. Additionally, the benefit of the similarity in the spectral response to (linear combinations of) NCMFs becomes more apparent when different lighting conditions, such as incandescent and white LED, are considered (see Supplementary Figure S9 for details). These results demonstrate that CAPs can achieve color reproduction similar to that captured by the naked human eye under various illumination sources.

To compare colors not on the Gretag-Macbeth color chart, we conducted color comparisons for virtual objects with various Gaussian-shaped spectral reflectances with different central wavelengths and full width at half maximum (FWHM) (see Supplementary Figure S10 for details) to demonstrate that our device operates effectively across various spectra, not only limited to the spectral reflectance of the Gretag-Macbeth color chart. This was performed without modifying the conversion matrices that were optimized using the color chart data. Notably, in the case of Gaussian-shaped spectral reflectance with an FWHM of 100 nm, our method demonstrated superior color reproduction compared to the conventional CIS with Bayer RGB color filters.

Finally, we conducted an additional numerical analysis to assess the device performance with respect to the input polarizations and incident angles (see Supplementary Figure S11 for details). Under normal incidence, the spectral absorptances of the CAPs demonstrated isotropic behavior and were independent of incident light polarization, as expected from the symmetry of the structures. For angled incidences, the device showed polarization-dependent behaviors; therefore, we averaged the response for different polarizations, as would indeed be the case under typical lighting conditions. It was observed that the color deviation ( $\Delta E_{00}^{*}$ ) compared to the normal incidence case remains <3.0 up to ±14° and <6.0 up to ±28°. Note that these numbers represent the color deviations at specified angles. In actual photography, a single pixel accepts the range of incident angles simultaneously; thus, the color deviation is averaged within such angles. Therefore, in practice, the usable angular range is even larger, and CAPs are suitable for most consumer imaging systems with F-numbers not less than ∼1.4.

## 3 Conclusions

We introduced CAPs whose spectral absorptances can be represented as linearly independent combinations of NCMFs, demonstrating their potential to advance color imaging technology. The proposed CAPs, with a compact design footprint featuring a device height of 860 nm and a pixel pitch of 221 nm, demonstrated strong absorption performances of 79 %, 81 %, and 63 % at wavelengths of 452 nm, 544 nm, and 603 nm, respectively. Furthermore, by intentionally designing CAPs whose spectral absorptances resemble linear combinations of NCMFs, our device accurately reproduced colors as captured by the naked human eye, exhibiting an average color difference ( $\Delta E_{00}^{*}$ ) of 1.79 when evaluated using the Gretag-Macbeth color chart under standard illuminant D65. Notably, the CAPs consistently show exceptional color fidelity under diverse illuminating light sources. Furthermore, we emphasize that the optical performance of the proposed CAPs can be further improved using an inverse design method, such as achieving CAPs with a single-layer structure while minimizing reflection and enhancing tolerance under oblique incidence. We believe that this technology has the potential to revolutionize the field of optical imaging sensors.

## 4 Methods

### 4.1 Simulation

We used a commercial finite-difference time-domain solver in ANSYS for the numerical simulations of the CAPs. The refractive indices of the materials were determined by ellipsometric measurements. In the unit cell, periodic boundary conditions were applied in the *x-* and *y*-directions, and perfectly matched layer absorbing boundary conditions were applied in the *z*-direction under linearly polarized incident light at various angles. The particle swarm optimization method was employed to optimize CAPs whose spectral absorptances can be represented as linear combinations of NCMFs [25]. The absorption profiles of the CAPs unit cell were calculated using ohmic loss, specifically $\frac{1}{2}\omega \varepsilon^{\prime \prime }{\left|E\right|}^{2}$ , where *ω* represents the angular frequency of the electromagnetic wave, and *ɛ*″ represents the imaginary part of the dielectric constant.

### 4.2 Possible sample preparation processes

SiO_2_ is deposited on a Si substrate by PECVD, followed by the deposition of polycrystalline Si on top of the SiO_2_ layer using the same technique. This layering process is repeated for the fabrication of DBR by depositing alternating layers of SiO_2_ and polycrystalline Si. Then, boron-ion implantation is conducted on the SiO_2_ surface for p-doping. Afterward, polycrystalline Si is deposited on the SiO_2_ layer using LPCVD, and a 50 nm SiO_2_ layer is deposited on the polycrystalline Si layer using PECVD, followed by arsenic-ion implantation for n-doping. Another layer of SiO_2_ is added on top using PECVD, with subsequent boron ion implantation for further p-doping. Polycrystalline Si is then deposited using LPCVD, covered by a 50 nm SiO_2_ layer through PECVD, and arsenic-ion implantation is performed for n-doping. Rapid thermal processing activates and diffuses the dopants into the polycrystalline Si layers, and the top SiO_2_ layer is etched away. An array of polycrystalline Si nanodisks, optimized in diameter for the CAPs structure, is created using e-beam lithography. Conformal SiO_2_ deposition fills the narrow gaps between nanodisk arrays using PECVD with a TEOS source. The excess SiO_2_ is trimmed using Ar plasma, and SiO_2_ is then deposited for surface planarization. The SiO_2_ is etched until a thickness of 30–50 nm remains above the nanodisk array. For electrical contacts, nanoholes are patterned on top of the n-doped Si nanodisk arrays, and ITO is sputtered to a thickness of 50 nm.

## Supplementary Material

Supplementary Material Details
