# Supplementary material for: Color arrestor pixels for high-fidelity, high-sensitivity imaging sensors
Source: Nanophotonics. 2024 Apr 15;13(16):2971–82. doi: 10.1515/nanoph-2024-0064 (PMC11502032; doi:10.1515/nanoph-2024-0064)
Supplement: Supplementary file 1 — Supplementary Material Details [file j_nanoph-2024-0064_suppl_001.docx]

**Supplementary Material for “Color arrestor pixels for high-fidelity, high-sensitivity imaging sensors”**

Mingwan Cho^1^, Joonkyo Jung^1^, Myungjoon Kim^1^, Jeong Yub Lee^2^, Seokhwan Min^1^, Jongwoo Hong^3^, Shinho Lee^3^, Minsung Heo^3^, Jong Uk Kim^3^, In-Sung Joe^3,*^ and Jonghwa Shin^1,^*

^1^ Department of Materials Science and Engineering, Korea Advanced Institute of Science and Technology (KAIST), Daejeon 34141, Republic of Korea.

^2^ Samsung Advanced Institute of Technology, 130, Samsung-ro, Yeongtong-gu, Suwon-si, Gyeonggi-do 16678, Republic of Korea.

^3^ Semiconductor Research Center, Samsung Electronics, Samsungjeonja-ro 1, Hwaseong-si, Gyeonggi-do, 18448, Republic of Korea.

*E-mail: [qubit@kaist.ac.kr](mailto:qubit@kaist.ac.kr), [insung.joe@samsung.com](mailto:insung.joe@samsung.com)

**
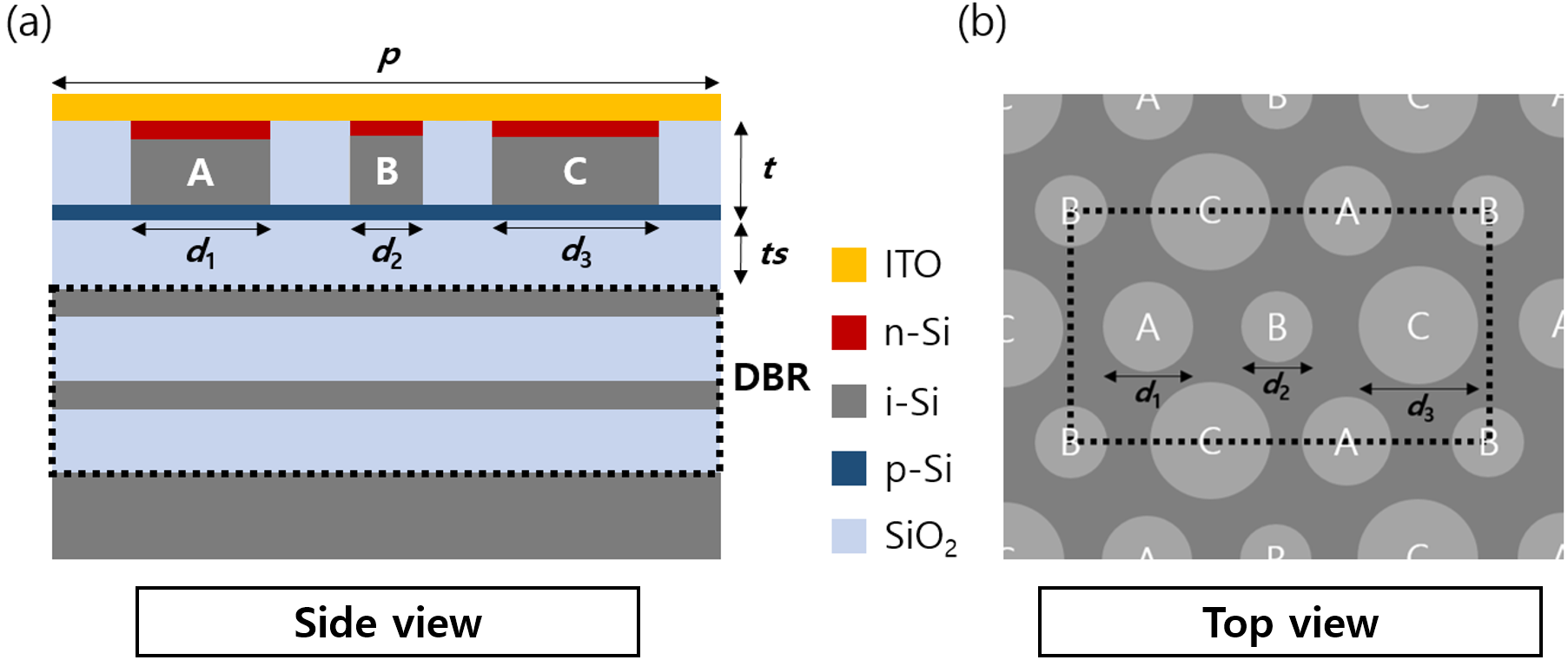
**


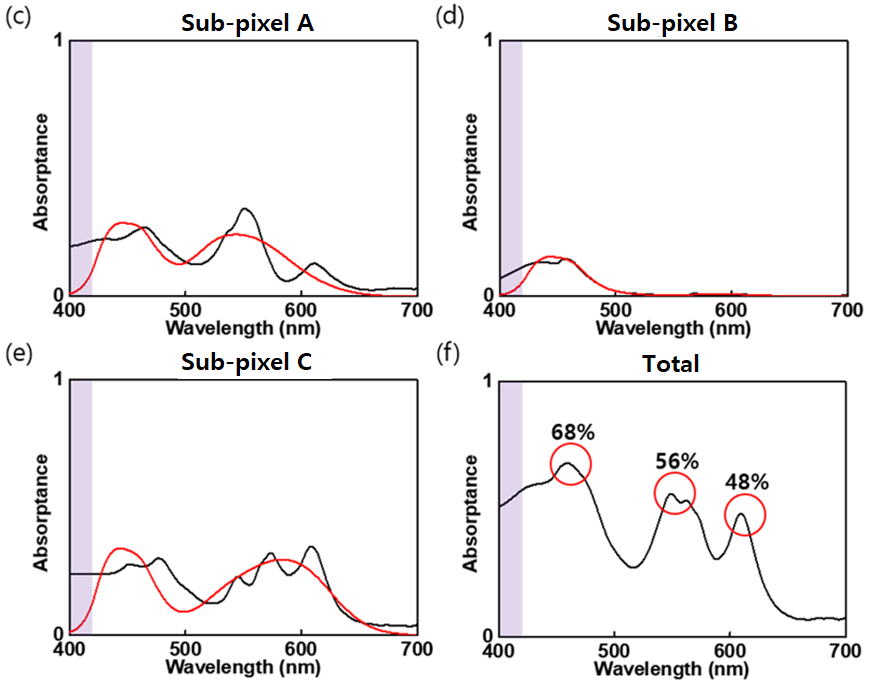


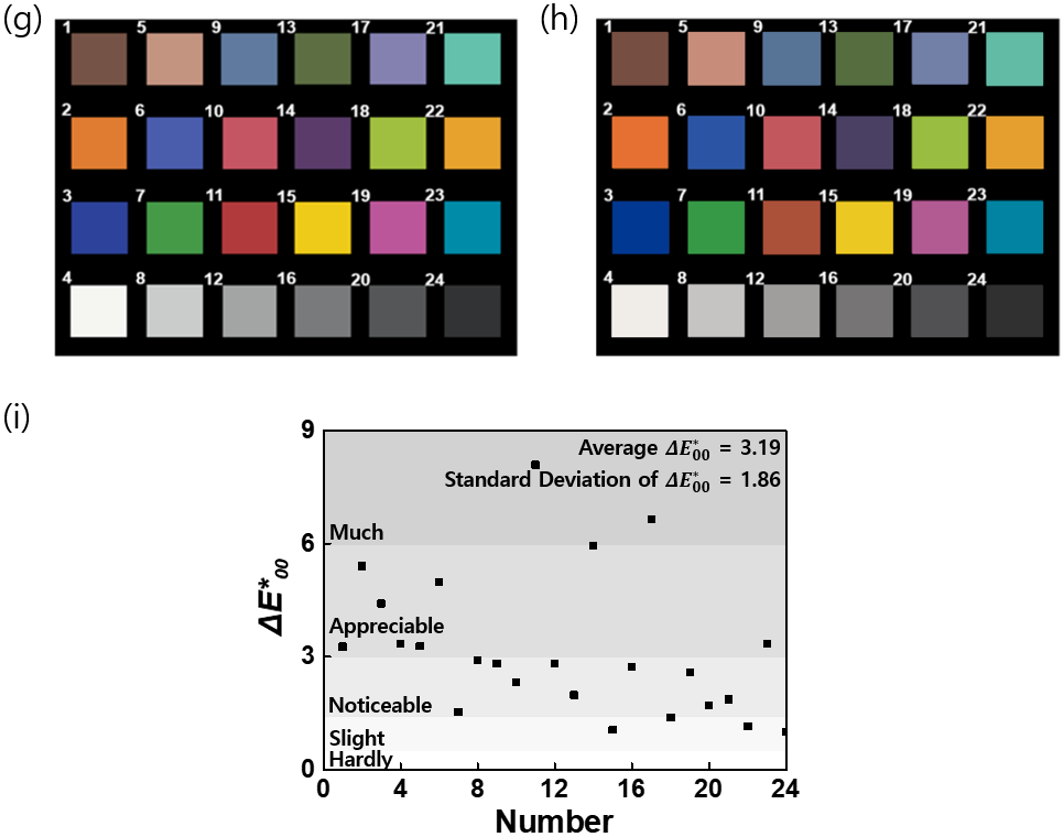


**Figure S1: Description of the single-layer CAPs.** (a) Side view of the single-layer CAPs unit cell design, consisting of Si nanodisk arrays with p-i-n junctions, including a SiO_2_ spacer layer on the DBR. (b) Top view of the single-layer CAPs design. The Si nanodisk arrays are arranged in a triangular lattice with intersecting positions, with each nanodisk responsible for individual sub-pixels. Additionally, the optimized values of the geometrical parameters were obtained as follows: $p$ = 300 nm, $t$ = 136 nm, $d_{1}$ = 177 nm, $d_{2}$ = 72 nm, $d_{3}$ = 200 nm, and $ts$ = 132 nm. (c)-(e) The spectral absorptances of single-layer CAPs are shown. The black solid line represents the simulated spectral absorptances $(A_{sim,A}(\lambda), A_{sim,B}(\lambda), A_{sim,C}(\lambda))$ of the optimized sub-pixels A, B, and C under linearly polarized light at normal incidence, obtained from finite-difference time-domain simulation and particle swarm optimization. The grey dashed line represents the curve-fitted spectral absorptances $(A_{fit,A}(\lambda),A_{fit,B}(\lambda), A_{fit,C}(\lambda))$, which are distinct linear combinations of the NCMFs resembling the simulated spectral absorptances $(A_{sim,A}(\lambda), A_{sim,B}(\lambda), A_{sim,C}(\lambda))$. The total spectral absorptance is shown in (f). The shaded region indicates the presence of an UV filter (400–420 nm) assumed to be located on top of the single-layer CAPs. (g) The Gretag-Macbeth color chart under standard illuminant D65 2° observer. **(**h) Image extraction from the Gretag-Macbeth color chart under standard illuminant D65 2° observer by virtual single-layer CAPs. (i) The color difference values $({\Delta E}_{00}^{*})$ for each of the 24 colors of the Gretag-Macbeth color chart by virtual single-layer CAPs.


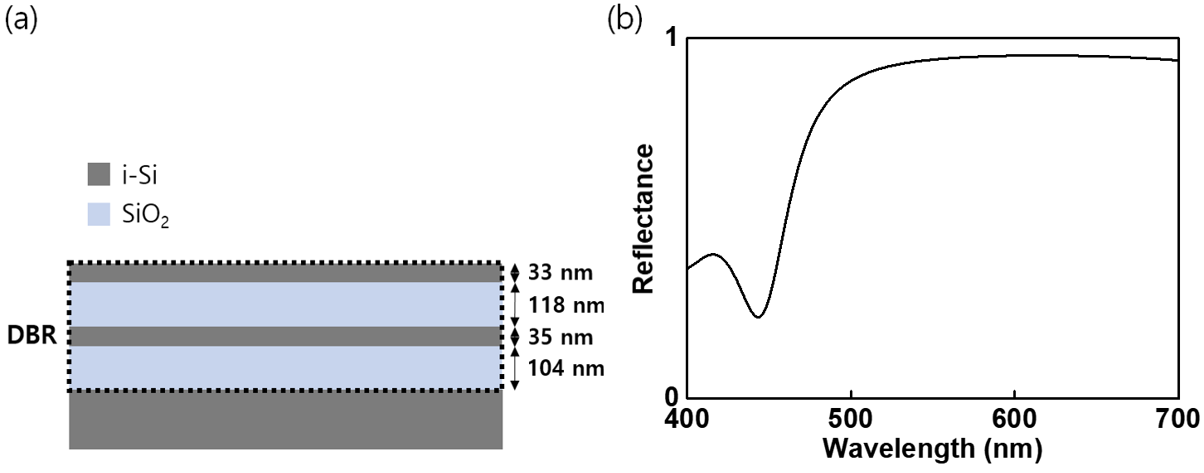


**Figure S2: Design of DBR.** (a) Schematic figure and (b) spectral reflectance of DBR.
The simulation was implemented to achieve high reflectance at the wavelength range of 500–700 nm. The DBR consists of alternating layers of SiO_2_ and Si, each with specific thicknesses: 104 nm, 35 nm, 118 nm, and 33 nm, as shown in Figure S2(a). The designed DBR achieves a high reflectance of 95% at the wavelength of 600 nm, as depicted in Figure S2(b).


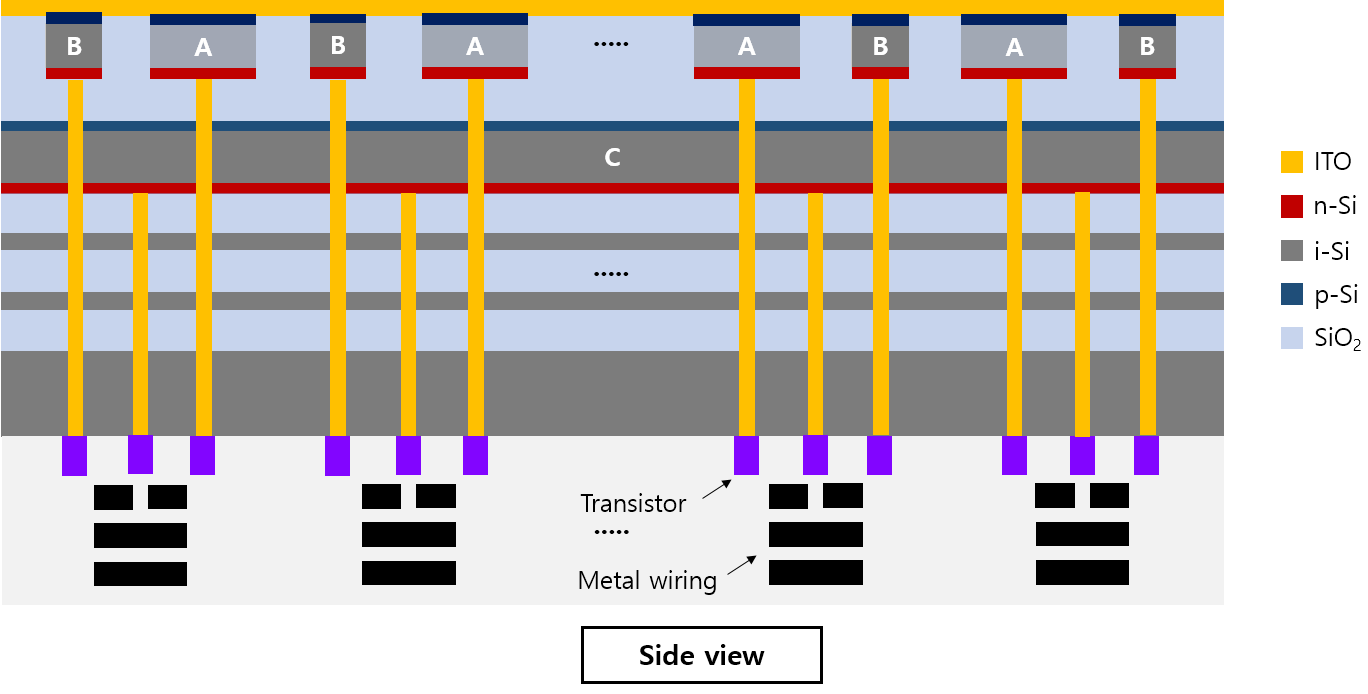


**Figure S3: Schematic figure of CAPs including electrical connection with CMOS readout circuit array.** For ease of individual pixel addressing, we reversed the positions of the n-doped and p-doped regions compared to the original CAPs design, which has negligible effect on the system’s optical responses. Each sub-pixel of the upper active layer shares a common electrical ground made of transparent ITO. The bottom of each sub-pixel of the upper active layer has individual ITO connections to the readout circuit below through vias, ensuring ohmic contact and enabling the desired current flow within the device. The bottom layer constituting sub-pixel C is also connected to the readout circuit through ITO vias. Each ITO via was assumed to be a cylinder with a diameter of 50 nm.


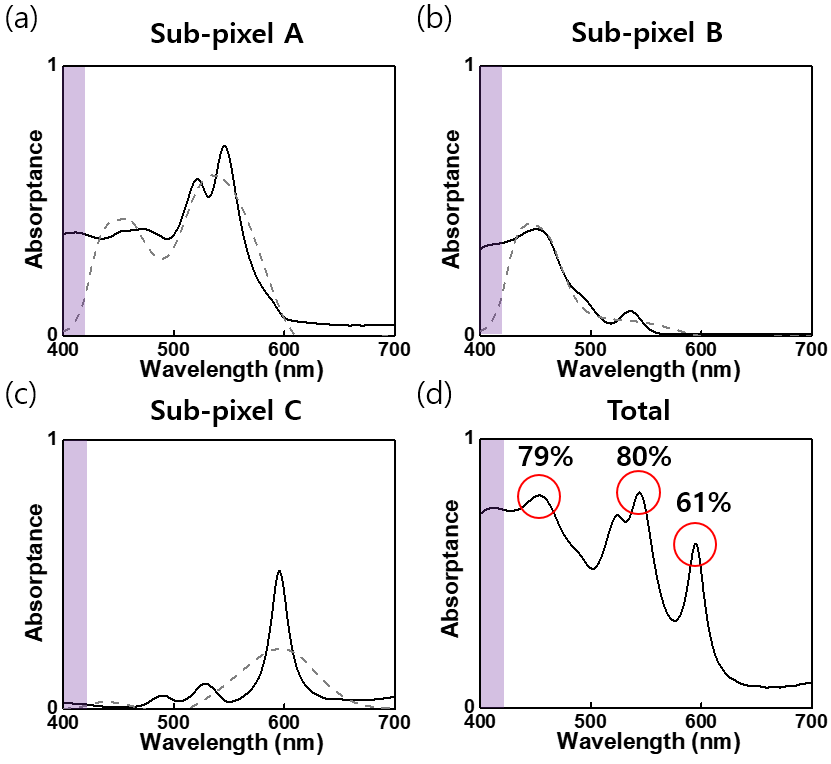


**Figure S4. Optical absorption characteristics of CAPs, featuring sub-pixels connected to metal wiring through ITO vias.** (a)-(c) The spectral absorptances of CAPs, featuring sub-pixels connected to metal wiring through ITO vias are shown. The black solid line represents the simulated spectral absorptances of the sub-pixels A, B, and C $(A_{sim,A}(\lambda), A_{sim,B}(\lambda), A_{sim,C}(\lambda))$ under linearly polarized light at normal incidence, obtained from finite-difference time-domain simulation and particle swarm optimization. The grey dashed line represents the curve-fitted spectral absorptances $(A_{fit,A}(\lambda),A_{fit,B}(\lambda), A_{fit,C}(\lambda))$, which are distinct linear combinations of the NCMFs resembling the simulated spectral absorptances $(A_{sim,A}(\lambda), A_{sim,B}(\lambda), A_{sim,C}(\lambda))$. The total spectral absorptance is shown in (d). The shaded region indicates the presence of an UV filter (400–420 nm) assumed to be located on top of the CAPs.

We added the ITO via electrodes and a back reflector mirror to represent metal wiring, without further optimizing the structural parameters of CAPs. We confirmed that such changes to the structure do not destroy important optical absorption features of CAPs as shown in Figure S4. With additional optimization of the structure considering the presence of the electrodes would further improve its performance. Indeed, individual pixel addressing at this length scale poses a significant challenge. This would be probably the reason why prior publications using metasurfaces in color filter-free CIS have not demonstrated individual addressing [1-4]. Although it remains a hard challenge within the academic domain and presents difficulties at the laboratory level, we believe the industry could potentially find a feasible solution. For example, advancements such as the Foveon X3 sensor and successful individual addressing in over 200 layers of NAND flash memory suggest that leveraging similar technologies might not be entirely out of reach. Acknowledging the complexity of individual pixel addressing, we speculate that a setup resembling Figure S3 could be a step towards realizing this goal.


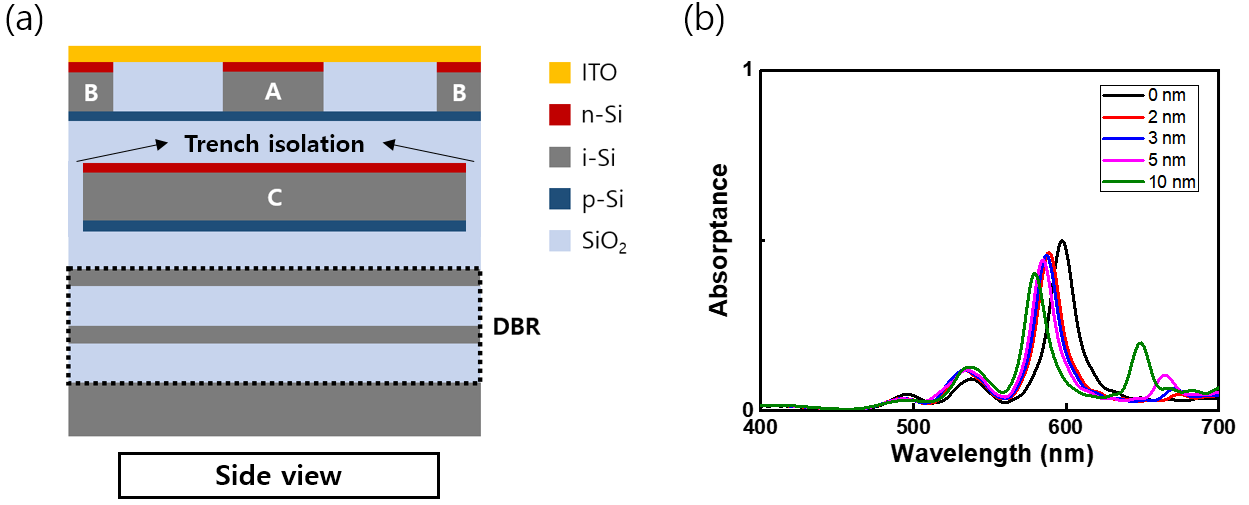


**Figure S5: Trench isolation structure.** (a) Schematic illustration and (b) spectral absorptances of the CAPs with varying trench widths.


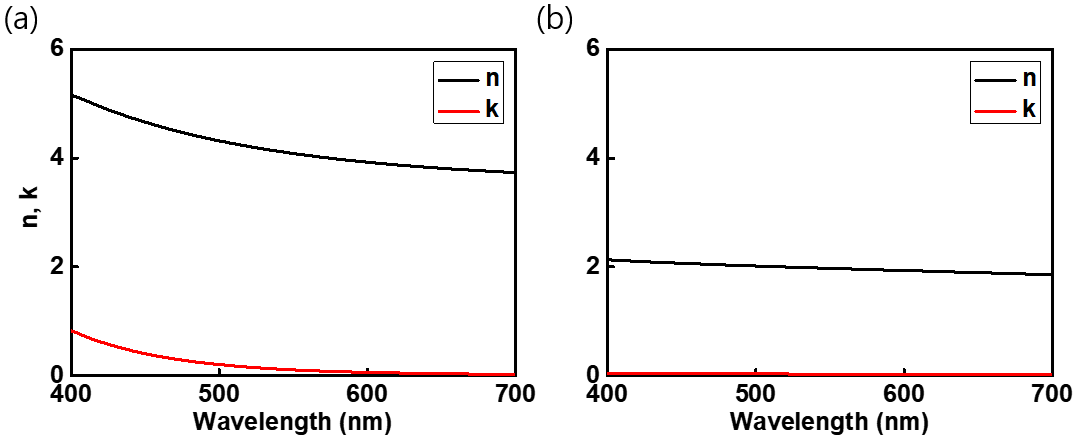


**Figure S6: Refractive index data.** Refractive index of (a) polycrystalline Si and (b) ITO. The refractive index spectra for polycrystalline Si and ITO were obtained through ellipsometry measurements, as shown in Figure S6(a) and (b).


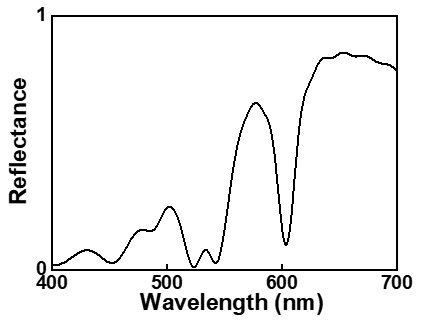


**Figure S7: Spectral reflectance of CAPs.** The CAPs exhibit clear reflection minima at the peak absorption wavelengths of 452 nm, 544 nm, and 603 nm, respectively.


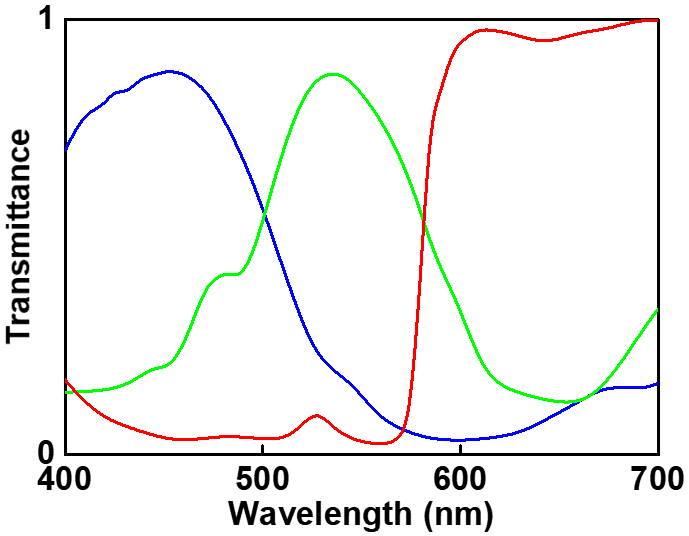


**Figure S8: Spectral transmittance of RGB color filters in conventional CIS.** This data was obtained from the pigment/dye filters of Fujifilm [5].


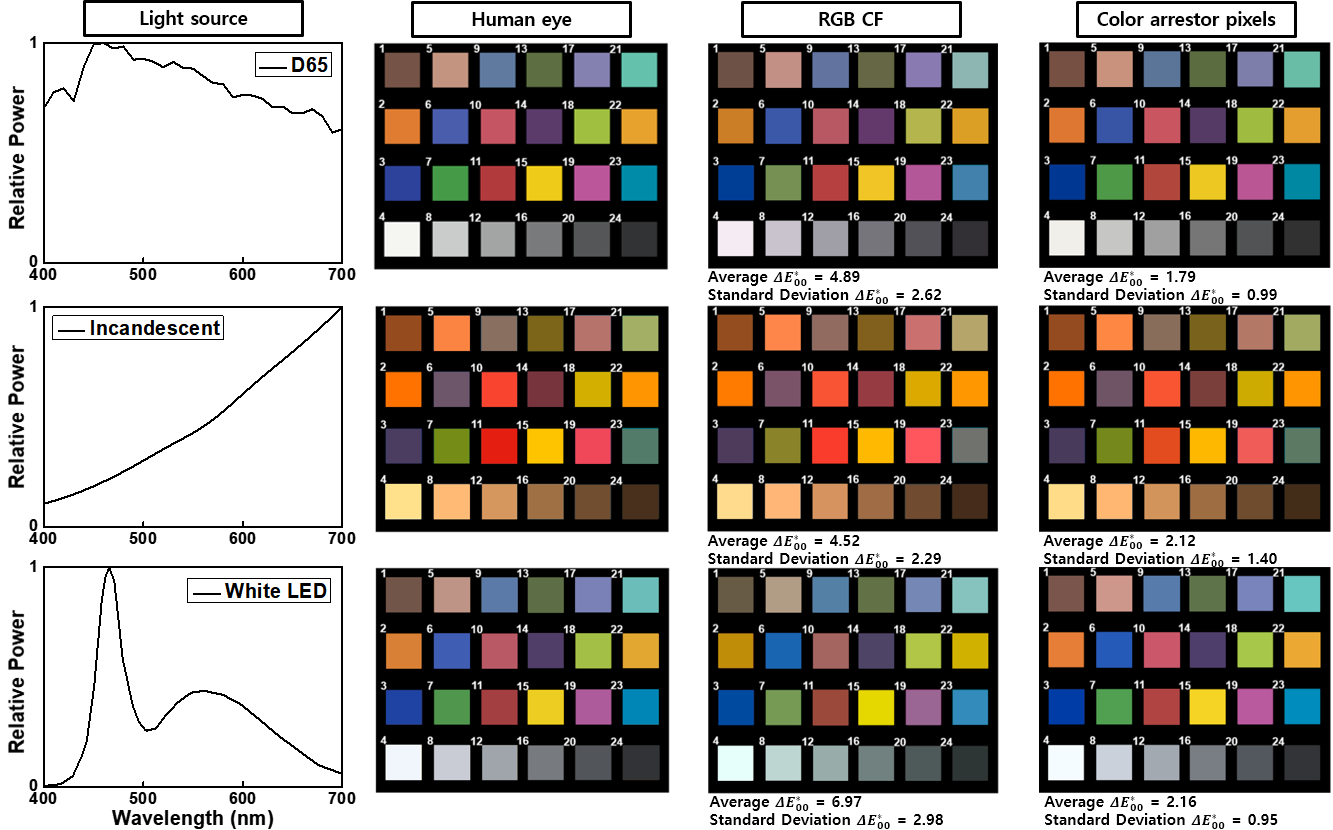


**Figure S9: Virtual images of the Gretag-Macbeth color chart under various illuminating light sources, captured by the naked human eye, CAPs, and conventional CIS.** The results demonstrate that the CAPs can achieve color reproduction similar to what the naked human eye captures under diverse illuminating light sources. This is achieved through the utilization of linearly independent combinations of NCMFs, enabling more accurate color reproduction than conventional CIS that uses RGB color filters.


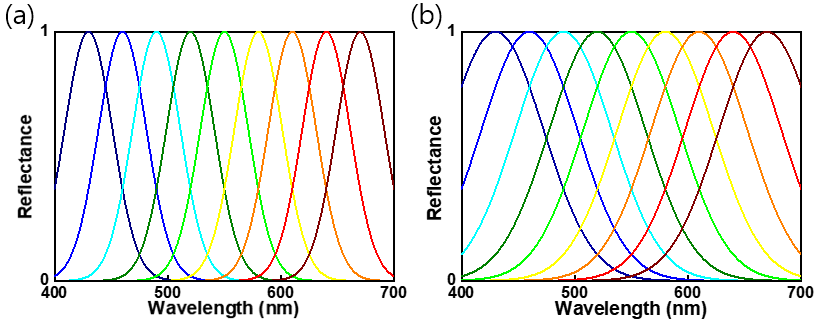


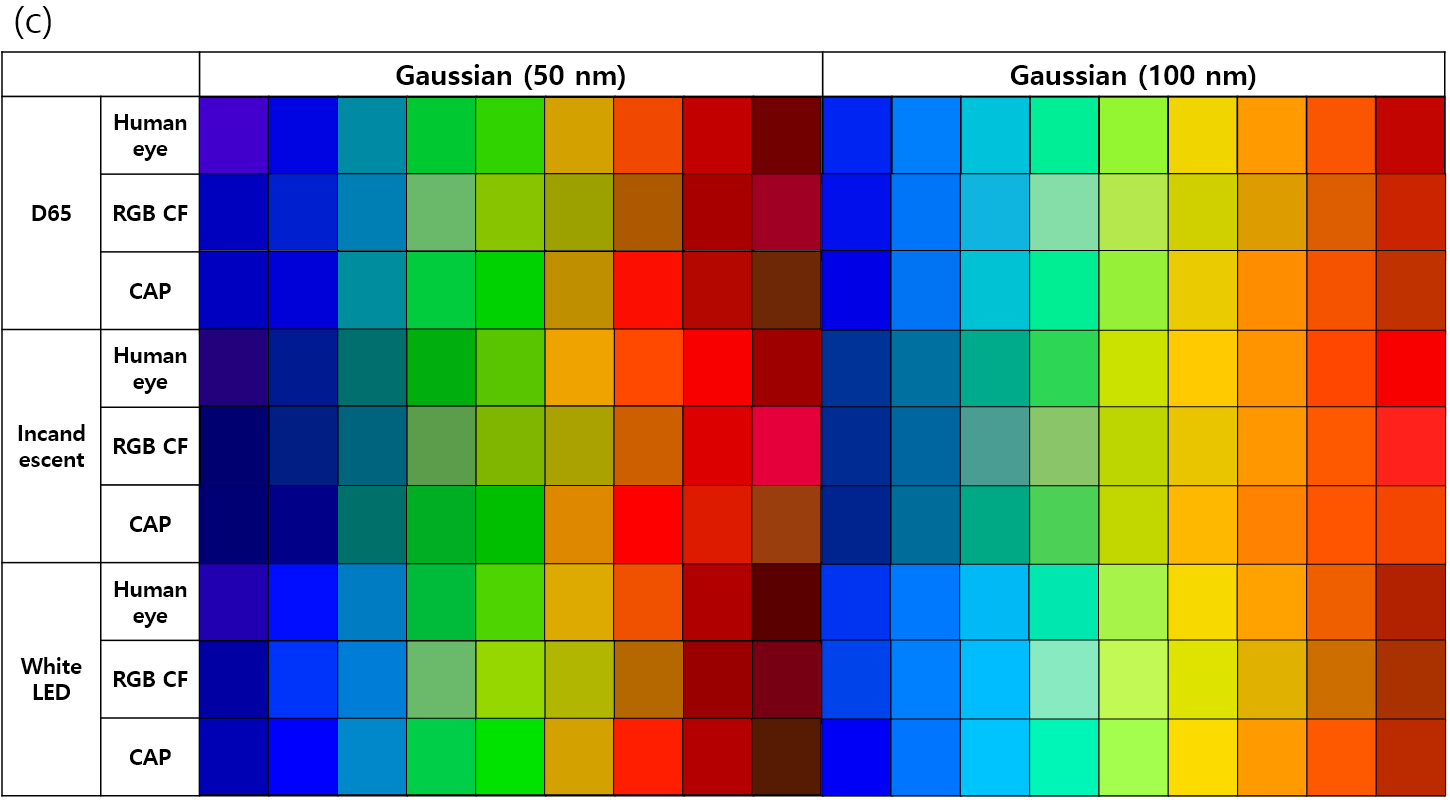


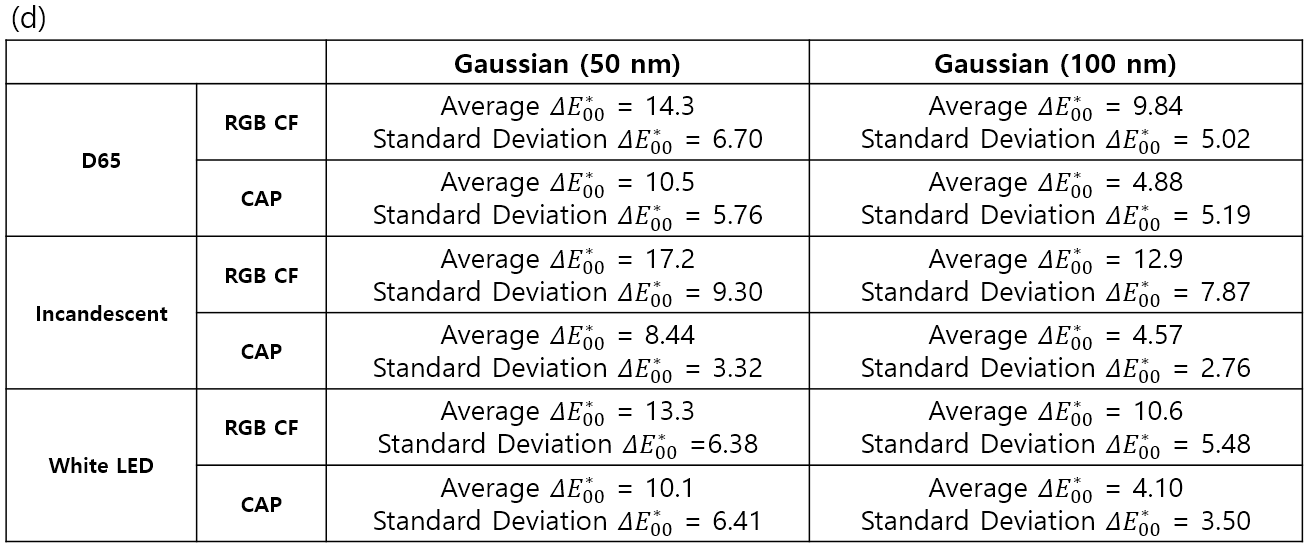


**Figure S10: Color comparisons for various Gaussian-shaped spectral reflectance with different central wavelengths and FWHM under various illuminating light sources.** (a) Nine spectral reflectance were assumed, starting from a central wavelength of 430 nm and increasing by 30 nm up to 670 nm. The FWHM was set at 50 nm. (b) Central wavelengths range from 430 nm to 670 nm with increments of 30 nm, resulting in nine spectral reflectance, all having a 100 nm FWHM. (c) Under light source such as standard illuminant D65, incandescent, and white LED, qualitative color comparisons were shown from the perspective of the naked human eye, conventional CIS based on RGB color filters, and CAPs for each of the spectral reflectance. (d) For each illuminating light sources and its respective spectral reflectance, the average color difference and standard deviation values were shown for conventional CIS with the RGB color filters and CAPs, comparing them to those observed by the naked human eye. For the spectral reflectance with a FWHM of 100 nm, CAPs exhibited superior accuracy of color reproduction compared to the conventional CIS with RGB color filters. In addition, for the case of a FWHM of 50 nm, CAP also showed good performance compared to the naked human eye.


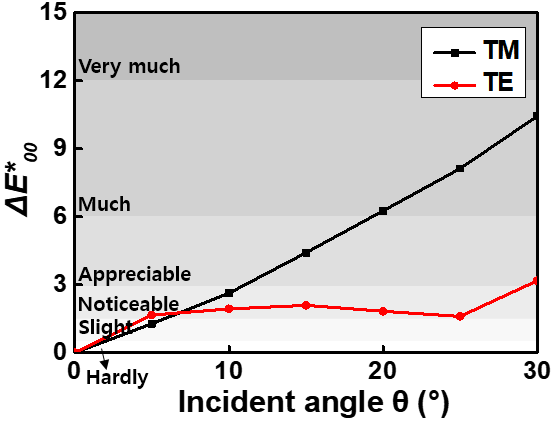


**Figure S11: Color difference values with respect to incident angle using CIEDE2000 from CIELAB.** In numerical simulations, light was incident at angles ranging from 0° to 30° in TE and TM waves. The spectral absorptances of CAPs exhibit isotropic properties, independent of the polarization state of normally incident light. However, differences in spectral absorptances are observed under the angled incidence conditions. We quantitatively analyzed the performance changes of CAPs based on incident angles using the CIEDE2000. Color difference values become noticeable when ${\Delta E}_{00}^{*}$ is less than 3 and appreciable when 3 < ${\Delta E}_{00}^{*}$ < 6 [6]. CAPs maintained ${\Delta E}_{00}^{*}$ < 3 for oblique incident angles less than ±11° for TM and greater than ±29° for TE mode incidence. In the analysis of color difference values under TE and TM wave incidences, it is evident that TM waves exert a more significant influence. The underlying reason for this is the interaction of the tangential electric field with electrons in the resonant wavelength range, leading to their oscillation. Moreover, as the angle of incidence for TM waves increases, there is a notable decrease in the effective wavelength of this electric field. For both TE and TM waves, the calculated color difference values become more pronounced with increasing incidence angles, attributed to the more confined interaction between the incident wave and the meta-atom. Furthermore, with greater incidence angles, there is a tendency for diffraction to happen at shorter wavelengths [7].

**Supplementary Note 1. Derivation process of the relationship formula between** $\boldsymbol{n}_{\mathbf{e}}$ **and** $\boldsymbol{A}\left( \boldsymbol{\lambda} \right)$

1. The power absorbed by CAPs, $P_{\mathrm{CAPs}}$ (unit : W)

$$\begin{aligned} P_{\mathrm{CAPs}}=P_{\mathrm{inc}}\int_{400}^{700} R\left( \lambda\right)I\left( \lambda\right)A\left( \lambda\right)d\lambda\#(4) \end{aligned}$$

$P_{\mathrm{inc}}$ : The power of incident light (unit : W)

$R\left( \lambda\right)$ : The spectral reflectance of an object

$I\left( \lambda\right)$ : The spectral power distribution of the illuminant (unit : $\frac{1}{nm}$)

$A\left( \lambda\right)$ : The spectral absorptance of the CAPs

2. The number of detected photons in the CAPs, $n_{p}$ (unit : $\frac{1}{s}$)

$$\begin{aligned} n_{p}=\frac{P_{\mathrm{CAPs}}}{h\nu}=P_{\mathrm{inc}}\int_{400}^{700} \frac{1}{h\nu}R\left( \lambda\right)I\left( \lambda\right)A\left( \lambda\right)d\lambda=P_{\mathrm{inc}}\int_{400}^{700} \frac{\lambda}{hc}R\left( \lambda\right)I\left( \lambda\right)A\left( \lambda\right)d\lambda=\int_{400}^{700} n_{p}^{'}\left( \lambda\right)d\lambda\#(5) \end{aligned}$$

$n_{p}^{'}\left( \lambda\right)$ : The spectral $n_{p}$ distribution (unit : $\frac{1}{nm*s})$

$h$ : The Planck constant (unit : $J*s$)

$\nu$ : The light frequency (unit : $\frac{1}{s}$)

3. The number of detected electrons in the CAPs, $n_{e}$ (unit : $\frac{1}{s}$)

$$\begin{aligned} n_{e}=\int_{400}^{700} n_{e}^{'}\left( \lambda\right)d\lambda=\int_{400}^{700} {\eta n}_{p}^{'}\left( \lambda\right)d\lambda=P_{\mathrm{inc}}\int_{400}^{700} \frac{\eta\lambda}{hc}R\left( \lambda\right)I\left( \lambda\right)A\left( \lambda\right)d\lambda\#(6) \end{aligned}$$

$n_{e}^{'}\left( \lambda\right)$ : The spectral $n_{e}$ distribution (unit : $\frac{1}{nm*s})$

$\eta$ : The internal quantum efficiency defined as the ratio between $n_{e}^{'}\left( \lambda\right)$ and $n_{p}^{'}\left( \lambda\right)$. We assumed that $\eta$ is equal to unity.

**Supplementary Note 2. Theoretical background**


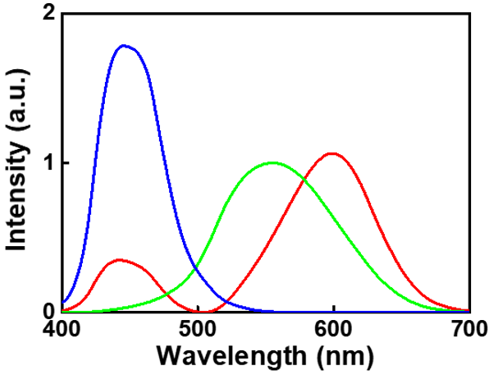


**Figure S12: The CIE 1931 standard observer CMFs,** $\bar{\boldsymbol{x}}\left( \boldsymbol{\lambda} \right)$**,** $\bar{\boldsymbol{y}}\left( \boldsymbol{\lambda} \right)$**, and** $\bar{\boldsymbol{z}}\left( \boldsymbol{\lambda} \right)$**.** The standardized CMFs provide a numerical representation of the chromatic response of the observer. The red, green, and blue solid lines represent $\bar{x}\left( \lambda\right)$, $\bar{y}\left( \lambda\right)$, and $\bar{z}\left( \lambda\right)$, respectively.

In the CIE 1931 color space, the color of an object with a specific spectral reflectance is typically expressed through tri-stimulus values denoted as ${(X}_{t}, Y_{t}, Z_{t})$. The equations are as follows:

$$X_{t}=\frac{1}{W}\int_{400}^{700} R\left( \lambda\right)I\left( \lambda\right)\bar{x}\left( \lambda\right)d\lambda$$

$$\begin{aligned} Y_{t}=\frac{1}{W}\int_{400}^{700} R\left( \lambda\right)I\left( \lambda\right)\bar{y}\left( \lambda\right)d\lambda\#(7) \end{aligned}$$

$$Z_{t}=\frac{1}{W}\int_{400}^{700} R\left( \lambda\right)I\left( \lambda\right)\bar{z}\left( \lambda\right)d\lambda$$

where $I(\lambda)$ refers to the spectral power distribution function of the illuminant (for example, standard illuminant D65, incandescent, and white LED), $R\left( \lambda\right)$ is the spectral reflectance (or transmittance) of an object, $\bar{x}\left( \lambda\right)$, $\bar{y}\left( \lambda\right)$, and $\bar{z}\left( \lambda\right)$ correspond to standardized CMFs as shown in Figure S12, $W$ is a scaling factor defined as $\int_{400}^{700} I\left( \lambda\right)\bar{y}(\lambda)d\lambda$, and $\lambda$ is the wavelength of the light. The spectral responsivity $S(\lambda)$ (unit : $\frac{A}{W}$) and the number of detected electrons in the CAPs $n_{e}$ (unit : $\frac{1}{s}$) are expressed as

$$\begin{aligned} S\left( \lambda\right)= \frac{q\eta\lambda}{hc}A\left( \lambda\right)\#(8) \end{aligned}$$

$$\begin{aligned} n_{e}=P_{\mathrm{inc}}\int_{400}^{700} \frac{\eta\lambda}{hc}R\left( \lambda\right)I\left( \lambda\right)A\left( \lambda\right)d\lambda= \frac{P_{\mathrm{inc}}}{q}\int_{400}^{700} R\left( \lambda\right)I\left( \lambda\right)S\left( \lambda\right)d\lambda\#(9) \end{aligned}$$

where $P_{\mathrm{inc}}$ is the power of incident light, $\eta$ refers to the internal quantum efficiency, which is assumed to be unity, $q$ is the charge of one electron, $h$ is the Planck constant, $c$ is the speed of light, and $A(\lambda)$ refers to the spectral absorptance of the intrinsic-Si region in sub-pixels. If the number of detected electrons in the CAPs ($n_{e})$corresponds to a linear combination of tri-stimulus values, the equation can be expressed as follows:

$$n_{e,A}= c_{11}X_{t}+c_{12}Y_{t}+c_{13}Z_{t}$$

$$\begin{aligned} n_{e,B}= c_{21}X_{t}+c_{22}Y_{t}+c_{23}Z_{t}\#(10) \end{aligned}$$

$$n_{e,C}= c_{31}X_{t}+c_{32}Y_{t}+c_{33}Z_{t}$$

The equation for the number of detected electrons in the CAPs ($n_{e})$ is shown in Eq. (9) and (10) can be merged as follows:

$$n_{e,A}=P_{\mathrm{inc}}\int_{400}^{700} \frac{\eta\lambda}{hc}R\left( \lambda\right)I\left( \lambda\right)A_{A}\left( \lambda\right)d\lambda= \frac{P_{\mathrm{inc}}}{q}\int_{400}^{700} {R\left( \lambda\right)I\left( \lambda\right)S}_{A}\left( \lambda\right)d\lambda= c_{11}X_{t}+c_{12}Y_{t}+c_{13}Z_{t}$$

$$\begin{aligned} n_{e,B}=P_{\mathrm{inc}}\int_{400}^{700} \frac{\eta\lambda}{hc}R\left( \lambda\right)I\left( \lambda\right)A_{B}\left( \lambda\right)d\lambda= \frac{P_{\mathrm{inc}}}{q}\int_{400}^{700} R\left( \lambda\right)I\left( \lambda\right)S_{B}\left( \lambda\right)d\lambda= c_{21}X_{t}+c_{22}Y_{t}+c_{23}Z_{t}\#(11) \end{aligned}$$

$$n_{e,C}=P_{\mathrm{inc}}\int_{400}^{700} \frac{\eta\lambda}{hc}R\left( \lambda\right)I\left( \lambda\right)A_{C}\left( \lambda\right)d\lambda= \frac{P_{\mathrm{inc}}}{q}\int_{400}^{700} {R\left( \lambda\right)I\left( \lambda\right)S}_{C}\left( \lambda\right)d\lambda= c_{31}X_{t}+c_{32}Y_{t}+c_{33}Z_{t}$$

In addition, the spectral responsivity $S(\lambda)$ is written as

$$S_{A}\left( \lambda\right)\boldsymbol{=}\frac{q}{P_{\mathrm{inc}}W}\left\{ c_{11}\bar{x}\left( \lambda\right)+ c_{12}\bar{y}\left( \lambda\right)+ c_{13}\bar{z}\left( \lambda\right) \right\}$$

$$\begin{aligned} S_{B}\left( \lambda\right)\boldsymbol{=}\frac{q}{P_{\mathrm{inc}}W}\left\{ c_{21}\bar{x}\left( \lambda\right)+ c_{22}\bar{y}\left( \lambda\right)+ c_{23}\bar{z}\left( \lambda\right) \right\}\#(12) \end{aligned}$$

$$S_{C}\left( \lambda\right)\boldsymbol{=}\frac{q}{P_{\mathrm{inc}}W}\left\{ c_{31}\bar{x}\left( \lambda\right)+ c_{32}\bar{y}\left( \lambda\right)+ c_{33}\bar{z}\left( \lambda\right) \right\}$$

The relationship between the spectral responsivity and the spectral absorptance is expressed by $S\left( \lambda\right)=\frac{q\eta\lambda}{hc}A(\lambda)$, so the spectral absorptances of CAPs are denoted by

$$A_{A}\left( \lambda\right)=\frac{hc}{\eta P_{\mathrm{inc}}W\lambda}(c_{11}\bar{x}(\lambda)+c_{12}\bar{y}(\lambda)+c_{13}\bar{z}(\lambda))$$

$$\begin{aligned} A_{B}\left( \lambda\right)=\frac{hc}{\eta P_{\mathrm{inc}}W\lambda}(c_{21}\bar{x}(\lambda)+c_{22}\bar{y}(\lambda)+c_{23}\bar{z}(\lambda))\#(13) \end{aligned}$$

$$A_{C}\left( \lambda\right)=\frac{hc}{\eta P_{\mathrm{inc}}W\lambda}(c_{31}\bar{x}(\lambda)+c_{32}\bar{y}(\lambda)+c_{33}\bar{z}(\lambda))$$

To simply and effectively represent the above equations as linear combination forms, we introduce new notations for the NCMFs ($\bar{x}'(\lambda), \bar{y}'(\lambda), \bar{z}'(\lambda))$. Illustrations of these functions are presented in Figure 2, and the formulas are as follows:

$$\bar{x}'(\lambda)=\frac{\lambda_{0}}{\eta\lambda}\bar{x}(\lambda)$$

$$\begin{aligned} \bar{y}'(\lambda)=\frac{\lambda_{0}}{\eta\lambda}\bar{y}(\lambda)\#(14) \end{aligned}$$

$$\bar{z}'(\lambda)=\frac{\lambda_{0}}{\eta\lambda}\bar{z}(\lambda)$$

By substituting Eq. (14) into Eq. (13), the simplified expressions are

$$A_{A}\left( \lambda\right)={\frac{hc}{P_{\mathrm{inc}}W\lambda_{0}}(c}_{11}\bar{x}'(\lambda)+c_{12}\bar{y}'(\lambda)+c_{13}\bar{z}'(\lambda))$$

$$\begin{aligned} A_{B}\left( \lambda\right)={\frac{hc}{P_{\mathrm{inc}}W\lambda_{0}}(c}_{21}\bar{x}'(\lambda)+c_{22}\bar{y}'(\lambda)+c_{23}\bar{z}'(\lambda))\#(15) \end{aligned}$$

$$A_{C}\left( \lambda\right)={\frac{hc}{P_{\mathrm{inc}}W\lambda_{0}}(c}_{31}\bar{x}'(\lambda)+c_{32}\bar{y}'(\lambda)+c_{33}\bar{z}'(\lambda))$$

Hence, If CAPs whose spectral absorptances correspond to the linear combinations of NCMFs ($\bar{x}'(\lambda), \bar{y}'(\lambda), \bar{z}'(\lambda))$ are designed, the values of the spectral responsivity $S(\lambda)$ and the number of detected electrons in the CAPs $(n_{e})$ can be determined. Furthermore, Eq. (10) can be represented in simple matrix form as

$$\begin{aligned} \left[ \begin{matrix} n_{e,A} \\ n_{e,B} \\ n_{e,C} \end{matrix} \right]=\left[ \begin{matrix} c_{11} & c_{12} & c_{13} \\ c_{21} & c_{22} & c_{23} \\ c_{31} & c_{32} & c_{33} \end{matrix} \right]\left[ \begin{matrix} X_{t} \\ Y_{t} \\ Z_{t} \end{matrix} \right]\#(16) \end{aligned}$$

If the row vectors of the coefficient matrix are linearly independent, the vector of the true tri-stimulus values ${(X}_{t}, Y_{t}, Z_{t})$ can be calculated by multiplying the inverse of the coefficient matrix using Eq. (16), which enables color representation. The matrix equation is expressed as

$$\begin{aligned} \left[ \begin{matrix} X_{t} \\ Y_{t} \\ Z_{t} \end{matrix} \right]=\left[ \begin{matrix} c_{11} & c_{12} & c_{13} \\ c_{21} & c_{22} & c_{23} \\ c_{31} & c_{32} & c_{33} \end{matrix} \right]^{-1}\left[ \begin{matrix} n_{e,A} \\ n_{e,B} \\ n_{e,C} \end{matrix} \right]\#(17) \end{aligned}$$

**Supplementary Note 3. Process of extracting tri-stimulus values from the simulated CAPs**

The spectral absorptances of the simulated CAPs are expressed as follows

$$A_{sim,A}\left( \lambda\right)={\frac{hc}{P_{\mathrm{inc}}W\lambda_{0}}(c}_{11}\bar{x}'(\lambda)+c_{12}\bar{y}'(\lambda)+c_{13}\bar{z}'(\lambda))+{err}_{A}\left( \lambda\right)$$

$$\begin{aligned} A_{sim,B}\left( \lambda\right)={\frac{hc}{P_{\mathrm{inc}}W\lambda_{0}}(c}_{21}\bar{x}'(\lambda)+c_{22}\bar{y}'(\lambda)+c_{23}\bar{z}'(\lambda))+{err}_{B}\left( \lambda\right)\#(18) \end{aligned}$$

$$A_{sim,C}\left( \lambda\right)={\frac{hc}{P_{\mathrm{inc}}W\lambda_{0}}(c}_{31}\bar{x}'(\lambda)+c_{32}\bar{y}'(\lambda)+c_{33}\bar{z}'(\lambda))+{err}_{C}\left( \lambda\right)$$

where the error term $err\left( \lambda\right)$ represents the error between $A_{\mathrm{sim}}\left( \lambda\right)$ and $A_{\mathrm{fit}}\left( \lambda\right)$. Furthermore, the vector representing the number of detected electrons in the simulated CAPs ${(n}_{e,sim,A}, n_{e,sim,B}, n_{e,sim,C})$ can be calculated by substituting ${(A}_{sim,A}, A_{sim,B}, A_{sim,C})$ from Eq. (18) into Eq. (9).

Next, to derive ${(X}_{\mathrm{sim}}, Y_{\mathrm{sim}}, Z_{\mathrm{sim}})$ vector, which is closer to the true tri-stimulus value ${(X}_{t}, Y_{t}, Z_{t})$ vector, we multiplied the 3 × 3 conversion matrix ($T$) and used the least squares method to find the optimal $T$ matrix (see Supplementary Note 4 for details). A simple representation of the conversion process is expressed as:

$$\begin{aligned} \left[ \begin{matrix} X_{t} \\ Y_{t} \\ Z_{t} \end{matrix} \right]\sim T\left[ \begin{matrix} n_{e,sim,A} \\ n_{e,sim,B} \\ n_{e,sim,C} \end{matrix} \right]\#(19) \end{aligned}$$

After determining the optimal $T$ matrix, we obtain ${(X}_{\mathrm{sim}}, Y_{\mathrm{sim}}, Z_{\mathrm{sim}})$ vector by multiplying $T$ matrix with the ${(n}_{e,sim,A}, n_{e,sim,B}, n_{e,sim,C})$ vector as follows:

$$\begin{aligned} \left[ \begin{matrix} X_{\mathrm{sim}} \\ Y_{\mathrm{sim}} \\ Z_{\mathrm{sim}} \end{matrix} \right]=T\left[ \begin{matrix} n_{e,sim,A} \\ n_{e,sim,B} \\ n_{e,sim,C} \end{matrix} \right]\#(20) \end{aligned}$$

**Supplementary Note 4. Conversion matrix**

We implemented a computational method to accurately reproduce the colors captured by the CAPs and conventional CIS equipped with RGB color filters [8]. This method relies on a conversion matrix approach, enabling the direct conversion of detected signals into tri-stimulus values, while minimizing potential errors. Mathematically, this is represented as $Y=TX$, where $Y$ corresponds to the vector of true tri-stimulus values ${(X}_{t}, Y_{t}, Z_{t})$ that we aim to achieve, $X$ represents the vector of the detected signals ${(n}_{e,A}, n_{e,B}, n_{e,C})$ of each pixel, and $T$ is a 3 × 3 conversion matrix. For a conventional CIS equipped with RGB color filters, the detected signal vectors are calculated using Eq. (9), assuming $A\left( \lambda\right)$ is equivalent to the spectral transmittance of each RGB color filter [5]. Initially, the determination of matrix $T$ involves solving it based on the known values of $X$ and $Y$ for the 24 colors in the Gretag-Macbeth color chart. However, 72 sets of data pairs were collected with three illuminating light sources (standard illuminant D65, incandescent, and white LED). The least squares method was used to construct a conversion matrix to minimize errors. Hence, the tri-stimulus values can be calculated by multiplying the obtained conversion matrix by the signal vectors from each pixel ${(n}_{e,A}, n_{e,B}, n_{e,C})$.

**Supplementary Note 5. Separate optimization of spectral absorptance and the conversion matrix for color accuracy**


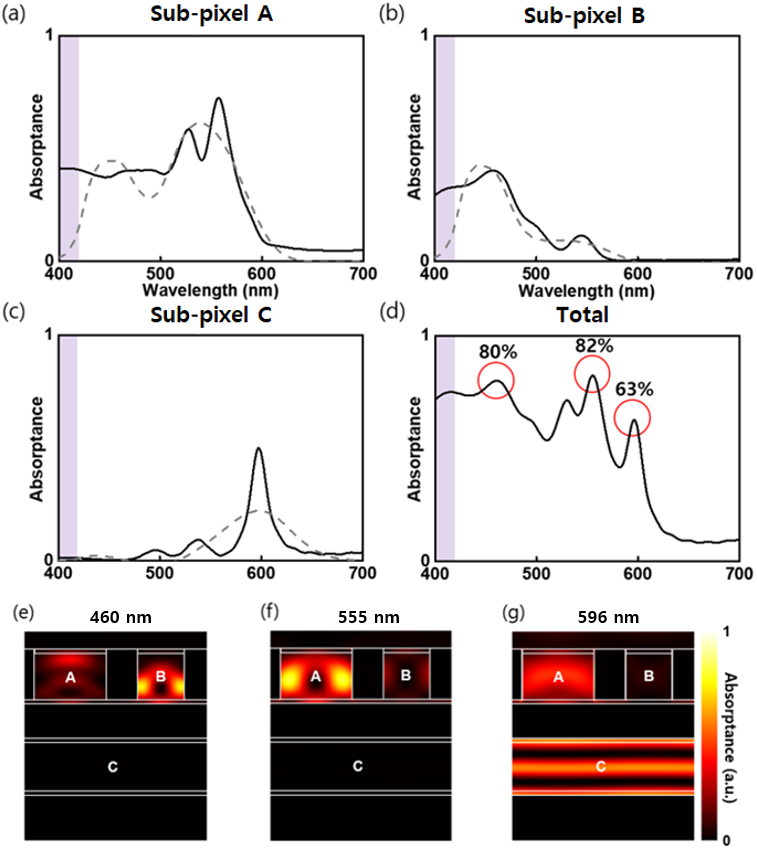


**Figure S13:** Numerical analysis of separately optimized CAPs. (a)-(c) The spectral absorptances of sub-pixels A, B, and C in the CAPs. The black solid lines represent the simulated spectral absorptances of sub-pixels A, B, and C $(A_{sim,A}(\lambda), A_{sim,B}(\lambda), A_{sim,C}(\lambda))$ under linearly polarized light at normal incidence, obtained from finite-difference time-domain simulation. The grey dashed lines represent the curve-fitted spectral absorptances of the sub-pixels A, B, and C $(A_{fit,A}(\lambda) , A_{fit,B}(\lambda) , A_{fit,C}(\lambda) )$, which are linear combinations of the NCMFs to resemble $(A_{sim,A}(\lambda), A_{sim,B}(\lambda), A_{sim,C}(\lambda))$. (d) The total spectral absorptance of the CAPs. The shaded region indicates the presence of an ultraviolet (UV) filter (400–420 nm) assumed to be located on top of the CAPs. (e-g) The normalized absorption profiles of the unit cell cross-section at the peak absorption wavelengths of 460 nm, 555 nm, and 596 nm, respectively.

We present the results of first optimizing the spectral absorptances of CAPs and then optimizing the conversion matrix to enhance color accuracy. The fundamental structure of the CAPs remains consistent with that shown in Figure 3. Initially, we defined the objective function $F$ for optimization according to Equation (3), which enabled us to derive the optimized geometrical parameter values as follows: $p$ = 220 nm, $t_{1}$ = 146 nm, $t_{2}$ = 152 nm, $d_{1}$ = 124 nm, $d_{2}$ = 80 nm, ${ts}_{1}$ = 95 nm, and ${ts}_{2}$ = 122 nm. In this configuration, Figures S13 (a-d) show the spectral absorptances of sub-pixels A, B, and C within the CAPs as well as the total spectral absorptance of the CAPs. Additionally, the normalized absorption profiles for the unit cell cross-section at the peak absorption wavelengths of 460 nm, 555 nm, and 596 nm are illustrated in Figures S13 (e-g), respectively.


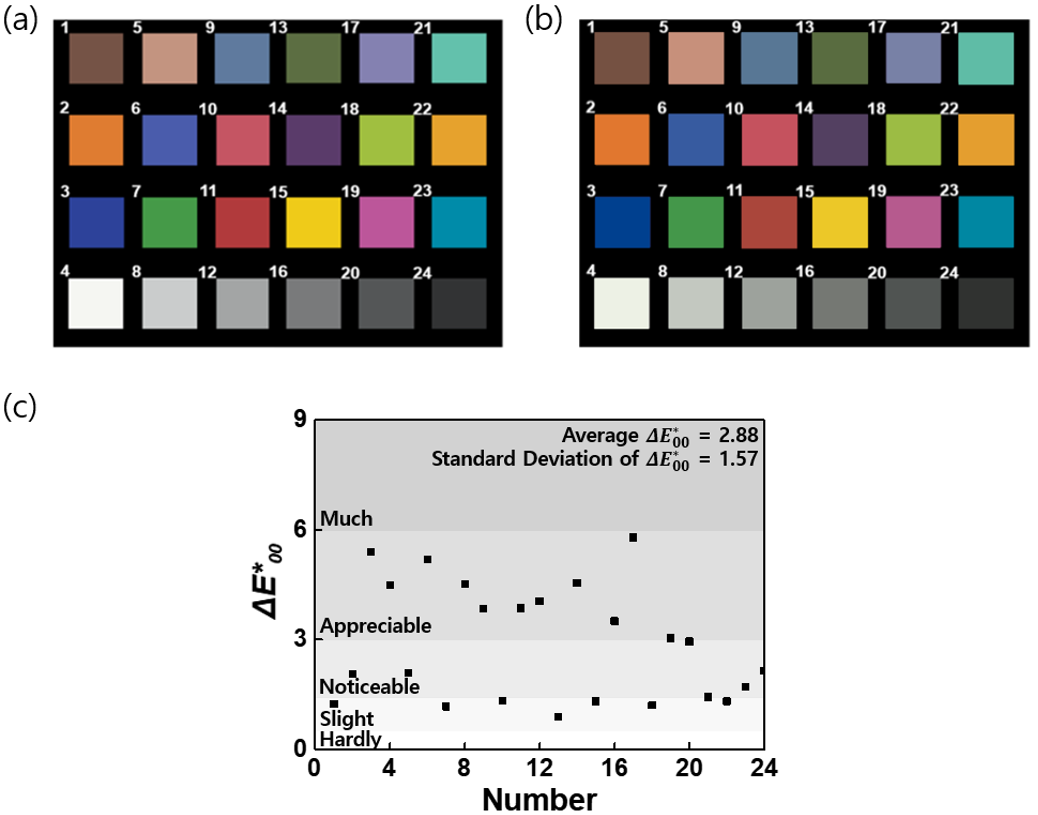


**Figure S14:** Color comparison. (a) The Gretag-Macbeth color chart under standard illuminant D65 2° observer captured by the naked human eye. (b) Image captured by separately optimized CAPs of the Gretag-Macbeth color chart under standard illuminant D65 2° observer. (c) The color difference values $({\Delta E}_{00}^{*})$ for each of the 24 colors of the Gretag-Macbeth color chart by separately optimized CAPs, employing the CIEDE2000 color difference metric.

After optimizing the spectral absorptances of each sub-pixel, an additional process was carried out to enhance color fidelity. We optimized a conversion matrix that transforms the output signals from the three sub-pixels of the CAPs into the tri-stimulus values. This was based on 72 cases, covering the 24 colors of the Gretag-Macbeth color chart under three different light sources: standard illuminant D65, incandescent, and white LED, as detailed in Supplementary Notes 3 and 4. In the case of separate optimization, the color image comparison from the Gretag-Macbeth color chart between the naked human eye and the CAPs under standard illuminant D65 2° observer is depicted in Figure S14 (a) and (b). The color difference values $({\Delta E}_{00}^{*})$ for each of the 24 colors of the Gretag-Macbeth color chart by CAPs is calculated in Figure S14 (c) and the average ${\Delta E}_{00}^{*}$ was 2.88 with a standard deviation of 1.57 under standard illuminant D65. In addition to that, the average ${\Delta E}_{00}^{*}$ was 2.45 with a standard deviation of 1.59 under incandescent and the average ${\Delta E}_{00}^{*}$ was 2.81 with a standard deviation of 1.33 under white LED as shown in Figure S15. Although separate optimization offers reasonably good performance, the CAPs designed through simultaneous optimization of the conversion matrix during structural optimization exhibit superior color reproduction in any other light source as shown in Figure S9.


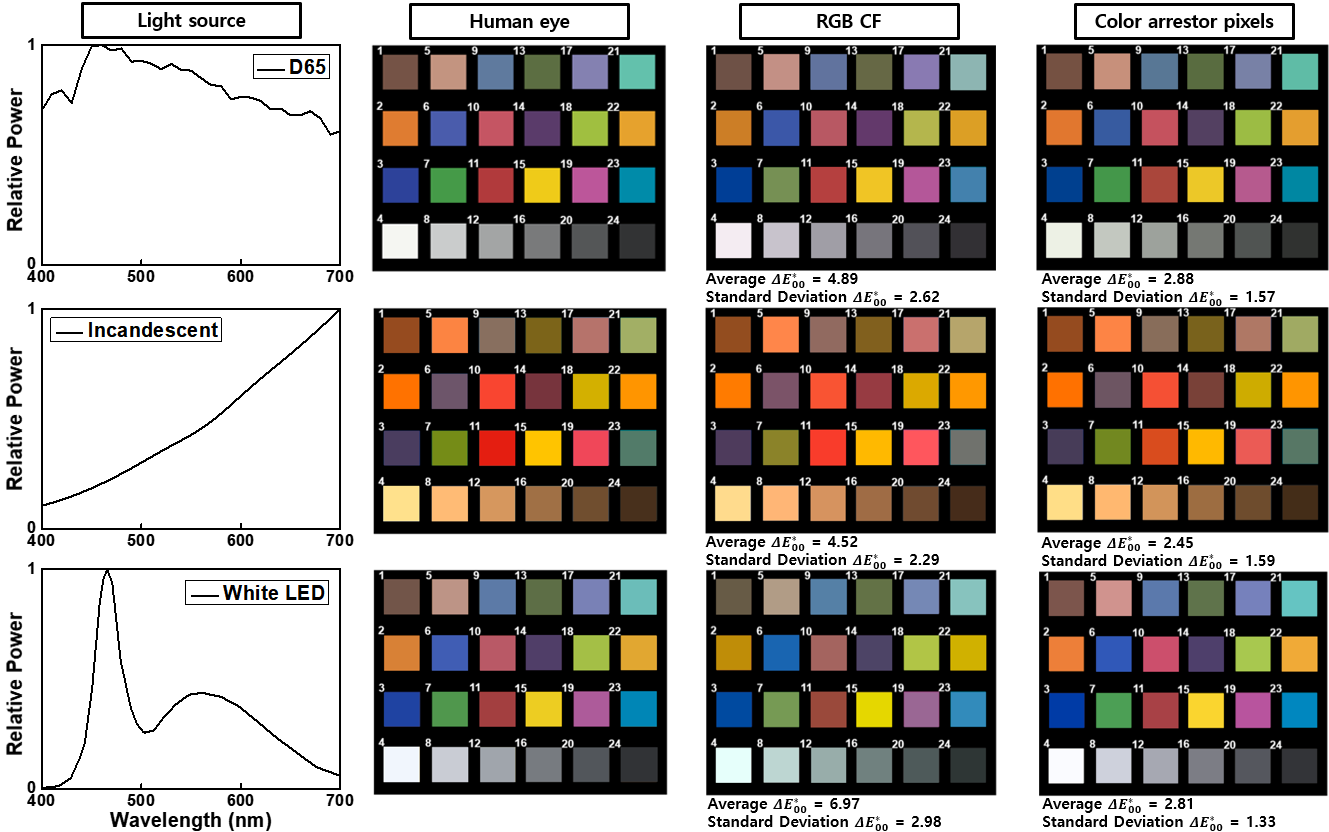


**Figure S15: Virtual images of the Gretag-Macbeth color chart under various illuminating light sources, captured by the naked human eye, separately optimized** **CAPs, and conventional CIS.** The results demonstrate that the separately optimized CAPs can achieve color reproduction similar to what the naked human eye captures under diverse illuminating light sources. This is achieved through the utilization of linearly independent combinations of NCMFs, enabling more accurate color reproduction than conventional CIS that uses RGB color filters.

**Supplementary Note 6. Physical description of the light absorption properties resulting from the designed sub-pixels of CAPs**

The resonant absorption in Si nanodisks of the upper layer in CAPs can be intuitively understood if we look at a simpler geometry with only one or a few disks on a substrate without other structures. One can place a plane wave source with perfectly matched layer (PML) boundaries in all directions as illustrated in Figure S16(a) and conduct FDTD simulations to measure the spectral absorptance of Si nanodisks. These nanodisks, corresponding to the diameter of sub-pixel A in the CAPs, were positioned on SiO_2_ substrate and varied in quantity of one, two, four, five, and nine units. As shown in Figure S16(b), the normalized spectral absorptance of a single Si nanodisk (A1) exhibited a substantial difference in the resonance peak location compared to those of sub-pixel A of CAPs. However, as the effect of array increased with more nanodisks, we observed a trend of decreasing difference in resonance peak locations. Similarly, analogous simulations were performed for sub-pixel B of CAPs using Si nanodisks of the same diameter, as depicted in Figure S16(c). While the trend was less pronounced compared to the Si nanodisk case for A, we confirmed a similar tendency of decreasing differences in resonance peak locations with an increasing number of nanodisks as shown in Figure S16(d).


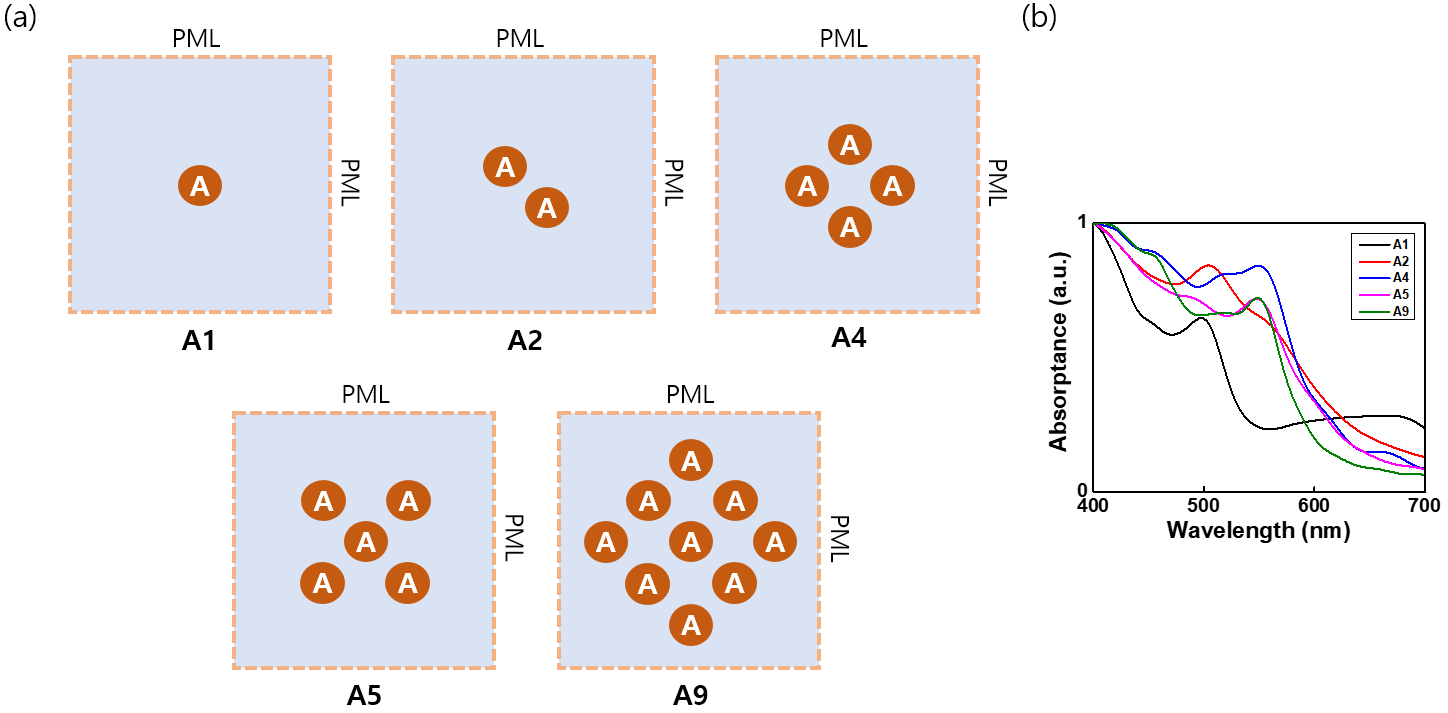


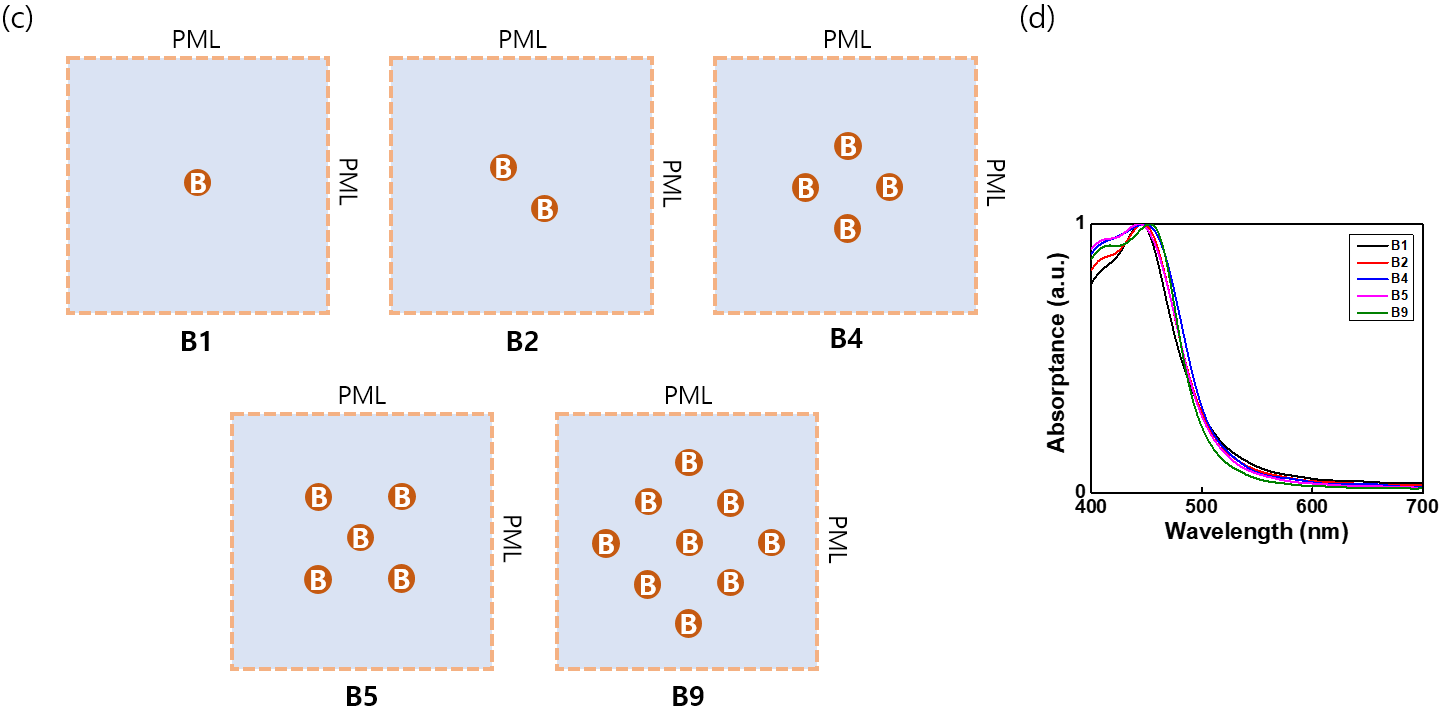


**Figure S16. Trends in normalized spectral absorptance for one, two, four, five, and nine Si nanodisks.** (a) Designs of Si nanodisks (one, two, four, five, and nine) with diameters identical to sub-pixel A of CAPs, placed within a PML boundary condition with a plain wave source. (b) Normalized spectral absorptance for one, two, four, five, and nine nanodisks of sub-pixel A. (c) Designs of one to nine Si nanodisks with diameters identical to sub-pixel B of CAPs, placed within a PML boundary condition with a plain wave source. (d) Normalized spectral absorptance for one, two, four, five, and nine nanodisks of sub-pixel B.

Next, we also conducted FDTD simulations on fully periodic arrays of silicon nanodisks, separately for types A and B, according to the original CAPs design. We set periodic boundary conditions and used a plane wave source. The designs for the separate arrays of A and B Si nanodisks, as well as the design for the C Si slab alone, are depicted in Figure S17(a)-(c). Their spectral absorptances were found not to significantly differ from those of the respective sub-pixels of the original CAPs, as shown in Figure S17(d)-(f). From these results, we infer that the observed spectral absorptance for sub-pixels A and B of CAPs likely originates from Mie resonance induced by the arranged Si nanodisks, while for sub-pixel C of CAPs, the observed effects likely arise from Fabry-Pérot resonance assisted absorption in the Si slab, influenced by the DBR layers positioned below.


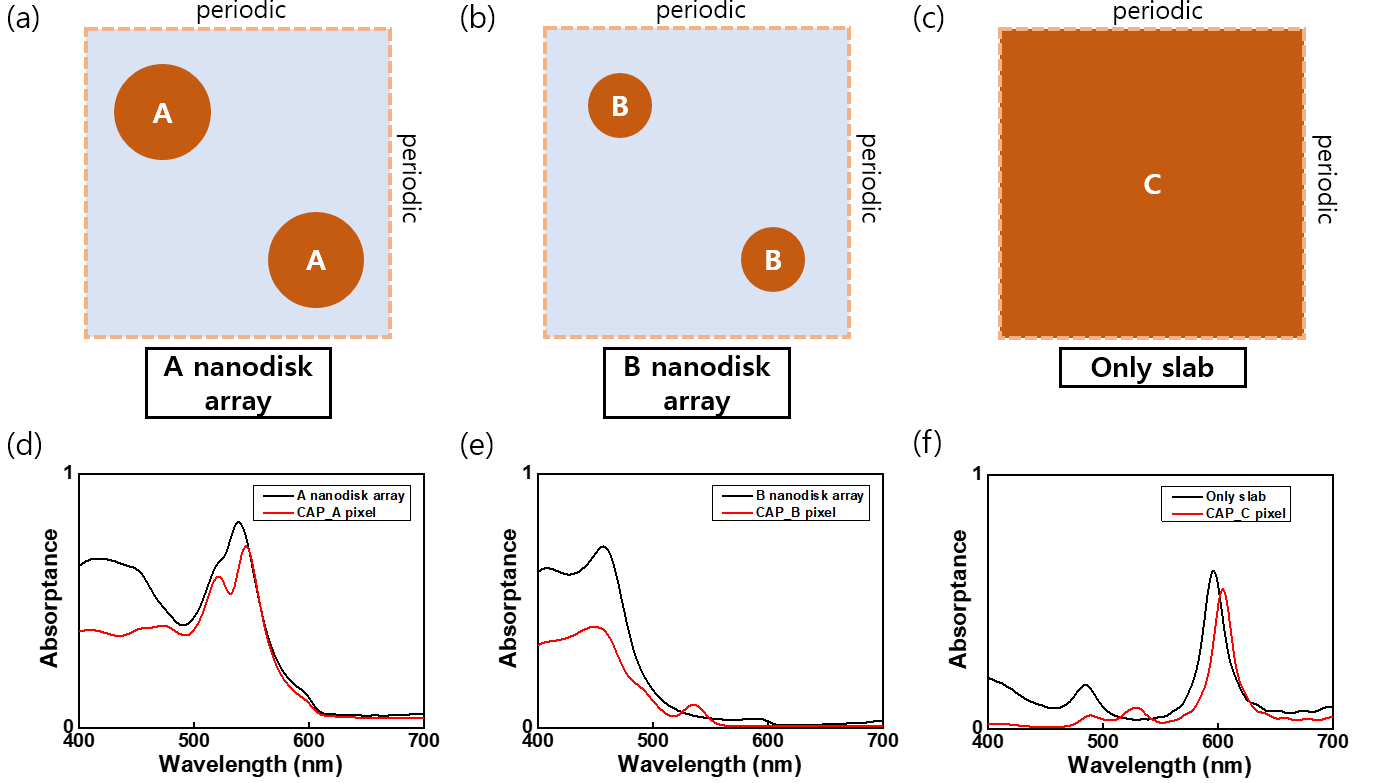


**Figure S17. Spectral absorptances for fully arranged Si nanodisks array for each type and for only C slab without nanodisk array in upper absorbing layer.** (a) Designs of Si nanodisks array with diameters identical to sub-pixel A of CAPs, placed within a periodic boundary condition with a plane wave source. (b) Designs of Si nanodisks array with diameters identical to sub-pixel B of CAPs, placed within a periodic boundary condition with a plane wave source. (c) Designs of only Si slab identical to sub-pixel C of CAPs, but without Si nanodisk array in the upper absorbing layer, positioned within periodic boundary conditions and subjected to a plane wave source. (d) Spectral absorptance for A nanodisk array (black solid line) and sub-pixel A of CAPs (red solid line). (e) Spectral absorptance for B nanodisk array (black solid line) and sub-pixel B of CAPs (red solid line). (f) Spectral absorptance for only Si slab (black solid line) and sub-pixel C of CAPs (red solid line).

**Supplementary Note 7. Flare consideration**

Strong light reflection above 600 nm in CAPs can lead to flare phenomena, common artifacts in photography in the presence of sun or other intense light sources. However, the effect may not be as severe as the reflectance numbers suggest. For instance, at a 650 nm wavelength, CAPs exhibit a high reflectance of 85% and a relatively low absorption rate of 8.4%, as depicted in Figures S7 and 4(d). In contrast, conventional CMOS image sensors (CIS) with RGB color filters, thanks to anti-reflective coatings, show much lower reflectance (below 10%) and, due to thicker Si photodetectors, significantly higher absorption (assumed as 30%). The crucial metric is the multiplication of reflectance and absorption rates, leading to an effective rate of 7.14% for CAPs versus 3.00% for RGB color filter-based CIS. Also, we note that such reflection and unwanted absorption appear across the entire spectral range in case of conventional CIS, whereas CAPs may face pronounced flare issues only at certain wavelengths. Thus, the total light energy re-entering the sensor due to reflection would be smaller than the above 7:3 ratio suggests, for typical white light sources. Using D65 as the light source, the ratio is close to 2:1 as calculated below. Additionally, lowering the cut-off wavelength of IR-cut filters down to 650 nm, instead of 700 nm, may further reduce this ratio to 7:4 and mitigate flare issues without significantly affecting color accuracy or sensor sensitivity of CAPs.

The following formula provides a simple quantitative model of the flare susceptibility, suggesting that, in practice, CAPs might not significantly worsen flare issues compared to conventional RGB color filter-based CIS, especially when considering the entire spectrum.

$$\begin{aligned} {Flare}_{\mathrm{CAP}}=\frac{\int_{400 nm}^{700 nm} D65(\lambda)\times R_{\mathrm{CAP}}(\lambda)\times R_{\mathrm{etc}}\times A_{\mathrm{CAP}}(\lambda)d\lambda}{\int_{400 nm}^{700 nm} D65(\lambda)\times A_{\mathrm{CAP}}(\lambda)d\lambda}=0.0193\#(21) \end{aligned}$$

$$\begin{aligned} {Flare}_{RGB-CF}=\frac{\int_{400 nm}^{700 nm} D65(\lambda)\times R_{RGB-CF}(\lambda)\times R_{\mathrm{etc}}\times A_{RGB-CF}(\lambda)d\lambda}{\int_{400 nm}^{700 nm} D65(\lambda)\times A_{RGB-CF}(\lambda)d\lambda}=0.01\#(22) \end{aligned}$$

where $D65(\lambda)$ is the standard illuminant, $R_{\mathrm{CAP}}(\lambda)$ is the spectral reflectance of CAPs, $R_{\mathrm{etc}}$ is the spectral reflectance of light reflected from lenses or other optical components (assumed as a constant value of 0.10), $A_{\mathrm{CAP}}(\lambda)$​ is the spectral absorptance of CAPs, $R_{RGB-CF}\left( \lambda\right)$is the spectral reflectance of RGB color filters based CIS (assumed as a constant value of 0.10), and $A_{RGB-CF}(\lambda)$ is the spectral absorptance of RGB-color filters based CIS. This formula enables us to estimate the ratio of the energy of photons that initially enter the highlight part of the sensor, get reflected by the sensor, and subsequently by other optical components, and finally reach again and are absorbed by the sensor to the energy of photons directly absorbed by the highlight area. Depending on the contrast between the highlight area and the dark area of the scene, a value of 0.01 may be noticeable. This gives us insight into the flare proneness of each device, and results suggest that in practical scenarios, CAPs may be two-times as prone to flares compared to RGB color filters based CIS. Lowering the IR-cut wavelength to 650 nm gives Flare_CAP_ and Flare_RGB-CF_ values of 0.0177 and 0.01.

**Supplementary Note 8. XYZ to sRGB conversion**

The XYZ color value $\left( X, Y, Z \right)$ vectors underwent a linear transformation to yield the sRGB value $\left( R, G, B \right)$ vectors. The matrix form is expressed as

$$\begin{aligned} \left[ \begin{matrix} R \\ G \\ B \end{matrix} \right]=\left[ \begin{matrix} 3.2406 & -1.5372 & -0.4986 \\ -0.9689 & 1.8758 & 0.0415 \\ 0.0557 & -0.2040 & 1.0570 \end{matrix} \right]\left[ \begin{matrix} X \\ Y \\ Z \end{matrix} \right]\#(23) \end{aligned}$$

After this transformation, gamma correction was applied to the $\left( R, G, B \right)$ vectors of each color channel to obtain the final sRGB values. This process adjusts the brightness and contrast of the colors. Gamma correction was performed as follows:

If the values of each $\left( R, G, B \right)$ are greater than 0.0031308, they are corrected as follows:

$$\begin{aligned} Value= 1.055 \times({Value}^{\frac{1}{2.4}}-0.055)\#(24) \end{aligned}$$

Otherwise, if the values of each $\left( R, G, B \right)$ were less than or equal to 0.0031308, they were corrected as follows:

$$\begin{aligned} Value= 12.92 \times Value\#(25) \end{aligned}$$

Finally, the corrected values were scaled to a range of 0–255, yielding 8-bit sRGB values.

**Supplementary Note 9. XYZ to CIE LAB conversion**

In contrast to the CIE 1931 color space, which is limited to chromaticity and presumes a linear perspective on human color perception, the CIELAB color space is more comprehensive. It encompasses the lightness $L^{*}$, as well as reflects the nonlinear nature of human visual perception. The chromaticity in this space is indicated by the parameters $a^{*}$ and $b^{*}$. The formulae are as follows:

$$L^{*}=116f\left( \frac{Y}{Y_{n}} \right)-16,$$

$$a^{*}=500\left( f\left( \frac{X}{X_{n}} \right)-f\left( \frac{Y}{Y_{n}} \right) \right),$$

$$\begin{aligned} b^{*}=200\left( f\left( \frac{Y}{Y_{n}} \right)-f\left( \frac{Z}{Z_{n}} \right) \right),\#(26) \end{aligned}$$

$$X_{n}=95.0489, Y_{n}=100, Z_{n}=108.8840,$$

$$\left( by standard illuminant D65 2^{\circ} observer \right)$$

$$X_{n}=110.629, Y_{n}=100, Z_{n}=41.3457,$$

$$\left( by illuminant Incandescent \right)$$

$$X_{n}=94.914, Y_{n}=100, Z_{n}=116.756,$$

$$(by illuminant White LED)$$

$$f\left( t \right)=\left\{ \begin{aligned} \sqrt[3]{t} if t> \delta^{3} \\ \frac{t}{3\delta^{3}}+\frac{4}{29} otherwise \end{aligned}, \right.\delta=\frac{6}{29}$$

where $\left( X_{n}, Y_{n}, Z_{n} \right)$ are the normalized reference values of the standard illuminant D65, incandescent, and white LED, respectively.

**Supplementary Note 10. CIEDE2000 color difference**

The color difference metric measures the extent of the difference in color perception between the two images. Conventionally, in color spaces, including CIERGB and CIELAB, the color difference is quantified using the Euclidean distance. Recognition of the limitations of the CIELAB space, stemming from the nonlinear nature of human color perception, led to the introduction of the CIEDE2000 formula. This method incorporates various correction and weighting factors for lightness, chroma, and hue, thereby facilitating a more precise assessment of color differences in a manner that aligns with human vision. The CIEDE2000 color difference is expressed by:

$$\begin{aligned} {\Delta E}_{00}^{*}=\sqrt{{(\frac{\Delta L'}{k_{L}S_{L}})}^{2}+{(\frac{\Delta C'}{k_{C}S_{C}})}^{2}+{(\frac{\Delta H'}{k_{H}S_{H}})}^{2}+R_{T}(\frac{\Delta C'}{k_{C}S_{C}})(\frac{\Delta H'}{k_{H}S_{H}})}\#(27) \end{aligned}$$

where $S_{L}, S_{C}$, and $S_{H}$ are the compensation terms for the lightness, chroma, and hue, respectively, in CIEDE2000. The values of $k_{L}, k_{C},$ and $k_{H}$ are compensation terms for the experimental conditions, and are typically set to unity. $R_{T}$ is the rotational correction factor. Detailed information on the calculation procedures for these compensation terms can be found in. [9].

**Supplementary Note 11. Sensor noise**

The increased sensitivity of CAPs helps color reproduction in the presence of noise as well (e.g., low-light photography). To address this question quantitatively, we considered readout noise and shot noise, which are commonly discussed as the primary sources of noise in the field of CMOS image sensors.

1) Readout noise

We set lux as the unit of illuminance, mirroring human visual perception. To convert power to lux, we utilized the following formula:

$$\begin{aligned} L= \frac{P_{\mathrm{inc}}\int_{400 \mathrm{nm}}^{700 \mathrm{nm}} I(\lambda)\times\bar{y}(\lambda)\times Kd\lambda}{(Sensor area)}\#(28) \end{aligned}$$

where $P_{\mathrm{inc}}$ is the power of incident light, $I(\lambda)$ is the normalized spectral power distribution function of the illuminant, $\bar{y}(\lambda)$ is the CIE photopic luminous efficiency function (equivalent to the color matching function, $\bar{y}(\lambda)$), and $K$ is the luminous efficacy, set at 683 lm/W for monochromatic light at 555 nm, the peak of human visual sensitivity. We assumed D65 as the illuminant and a sensor area of 75 mm^2^, equivalent to a 1/1.28 inch CMOS image sensor. We also assumed a readout noise of 3 electrons per pixel with a total pixel count of 100 million and applied a random Gaussian variable method to the total readout noise. We added the same readout noise to the number of electrons extracted from both conventional CIS with RGB color filters and CAPs. For illumination levels of 100 lux (very dark day), 10 lux (twilight), 1 lux (deep twilight), and 0.1 lux (night scene under full moon), we computed the average color difference for the 24 patches of the Gretag-Macbeth color chart. These calculations were performed over 100 trials for each illumination condition, and the mean outcomes were quantitatively presented in Table S1.


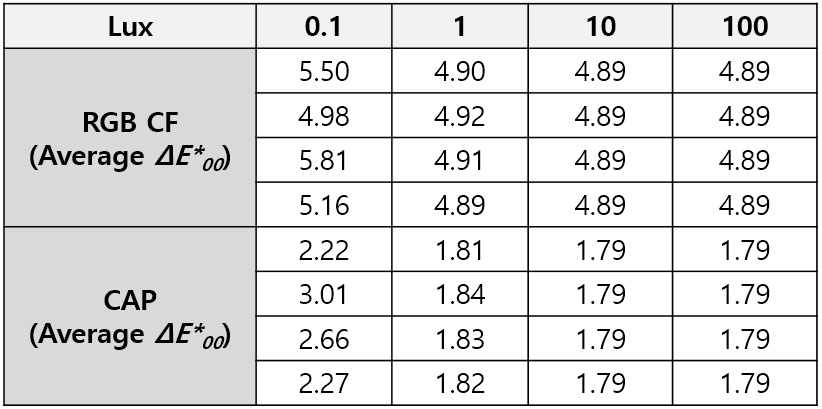


**Table S1. Comparisons of average color difference values with readout noise at different lux values.**

CAPs maintains superior color reproduction across all lux levels considered, compared to RGB color filter based CMOS image sensors. The increased light-detection capacity, especially around the important wavelength ranges, reduces the impact of readout noise, allowing CAPs to produce clearer images and high color accuracy in low-light conditions.

2) Shot noise

For addressing shot noise, we applied the Poisson distribution of the photon numbers to the incident light assuming illuminant D65’s spectral profile. Using this method, we re-evaluated the number of electrons extracted from both traditional CIS with RGB color filters and CAPs. For ease of calculation, we used wavelength integration bins of 1 nm intervals between 400nm and 700nm. We again evaluated the average color difference across the 24 patches of the Gretag-Macbeth color chart over 100 trials each for each illumination level. The mean results of these calculations were then quantitatively summarized in Table S2 for a clear comparison.


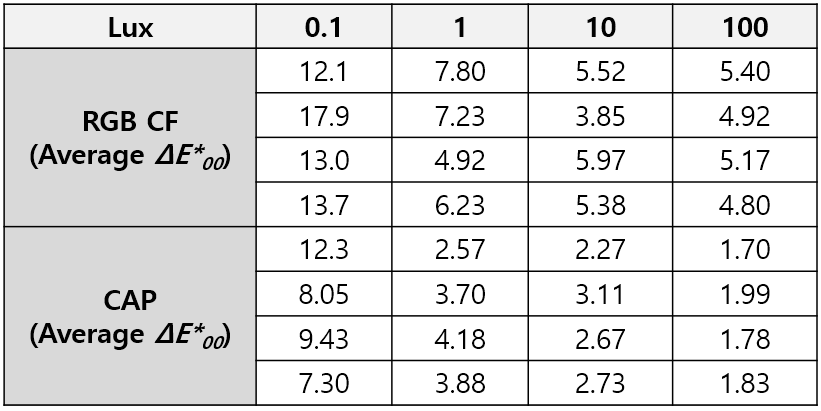


**Table S2. Comparisons of average color difference values with shot noise at different lux values.**

Here as well, CAPs demonstrates superior color reproduction across all lux levels considered.


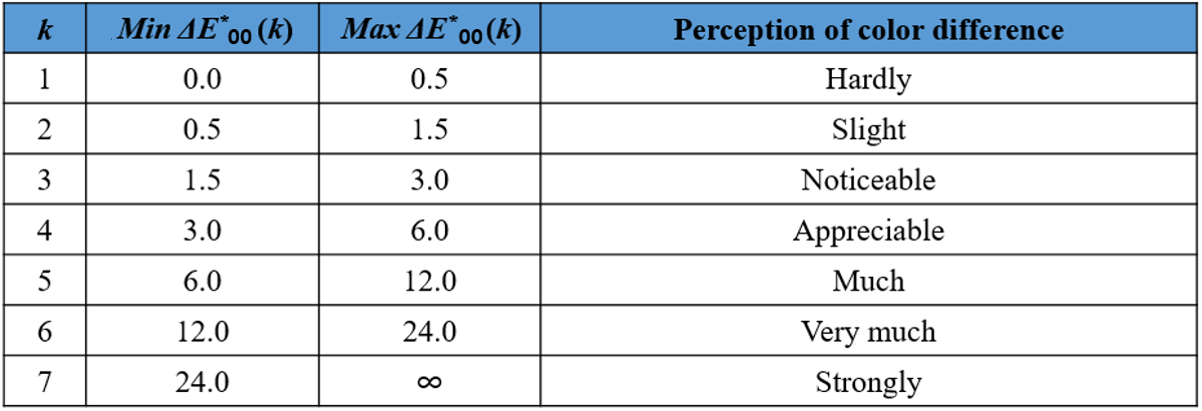


**Table S3:** Subjective assessment metric based on CIEDE2000 color difference.

**References**

1. J. S. T. Smalley, et al., “Subwavelength pixelated CMOS color sensors based on anti-Hermitian metasurface,” Nat. Commun., vol. 11, no. 1, 2020, Art. no. 3916, https://doi.org/10.1038/s41467-020-17743-y.
2. S. Kim, C. Lee, S. Jeon, J. Park, and S. Kim, “Subwavelength sorting of full-color based on anti-Hermitian metasurfaces,” Nanophotonics, vol. 10, no. 2, pp. 967–974, 2020. https://doi.org/10.1515/nanoph-2020-0526.
3. J. Ho, et al, “Miniaturizing color-sensitive photodetectors via hybrid nanoantennas towards sub-micron dimensions,” Sci. Adv., vol. 8, no. 47, p. eadd3868, 2022. https://doi.org/10.1126/sciadv.add3868.
4. H. Park, et al, “Filter-free image sensor pixels comprising silicon nanowires with selective color absorption,” Nano Lett., vol. 4, no. 4, pp. 1804–1809, 2014. https://doi.org/10.1021/nl404379w.
5. K. Sukeno, “Primary color filters for image sensor (IS) applications,” Fujifilm. Available at: https://www.fujifilm.com/us/en/business/semiconductor-materials/image-sensor-color mosaic/rgb/applications/ Accessed: Nov. 3, 2023.
6. Y. Yang, J. Ming, and N. Yu, “Color image quality assessment based on CIEDE2000,” Adv. Multimed., vol. 2012, p. 11, 2012. https://doi.org/10.1155/2012/273723.
7. J. Hong, H. Son, C. Kim, S.-E. Mun, J. Sung, and B. Lee, “Absorptive metasurface color filters based on hyperbolic metamaterials for a CMOS image sensor,” Opt. Express, vol. 29, no. 3, pp. 3643–3658, 2021. https://doi.org/10.1364/oe.415874.
8. M. Miyata, M. Nakajima, and T. Hashimoto, “High-sensitivity color imaging using pixel-scale color splitters based on dielectric metasurfaces,” ACS Photonics, vol. 6, no. 6, pp. 1442–1450, 2019. https://doi.org/10.1021/acsphotonics.9b00042.
9. M. R. Luo, G. Cui, and B. Rigg, “The development of the CIE 2000 colour-difference formula: CIEDE2000,” Color Res. Appl., vol. 26, no. 5, pp. 340–350, 2001. https://doi.org/10.1002/col.1049.
